# Haematological and biochemical reference intervals for wild green turtles (*Chelonia mydas*): a Bayesian approach for small sample sizes

**DOI:** 10.1093/conphys/coac043

**Published:** 2022-07-07

**Authors:** Sara Kophamel, Donna Rudd, Leigh C Ward, Edith Shum, Ellen Ariel, Diana Mendez, Jemma Starling, Renee Mellers, Richard K Burchell, Suzanne L Munns

**Affiliations:** College of Public Health, Medical and Veterinary Sciences, James Cook University, Townsville, Queensland, 4811, Australia; College of Public Health, Medical and Veterinary Sciences, James Cook University, Townsville, Queensland, 4811, Australia; School of Chemistry and Molecular Biosciences, The University of Queensland, Brisbane, Queensland, 4072, Australia; College of Public Health, Medical and Veterinary Sciences, James Cook University, Townsville, Queensland, 4811, Australia; College of Public Health, Medical and Veterinary Sciences, James Cook University, Townsville, Queensland, 4811, Australia; Australian Institute of Tropical Health and Medicine, James Cook University, Townsville, Queensland, 4811, Australia; College of Public Health, Medical and Veterinary Sciences, James Cook University, Townsville, Queensland, 4811, Australia; College of Public Health, Medical and Veterinary Sciences, James Cook University, Townsville, Queensland, 4811, Australia; North Coast Veterinary Specialist and Referral Centre, Sunshine Coast, Queensland, 4556, Australia; College of Public Health, Medical and Veterinary Sciences, James Cook University, Townsville, Queensland, 4811, Australia

**Keywords:** wildlife health, sea turtles, population assessment, blood analysis, baseline values, Australia

## Abstract

Animal health is directly linked to population viability, which may be impacted by anthropogenic disturbances and diseases. Reference intervals (RIs) for haematology and blood biochemistry are essential tools for the assessment of animal health. However, establishing and interpreting robust RIs for threatened species is often challenged by small sample sizes. Bayesian predictive modelling is well suited to sample size limitations, accounting for individual variation and interactions between influencing variables. We aimed to derive baseline RIs for green turtles (*Chelonia mydas*) across two foraging aggregations in North Queensland, Australia, using Bayesian generalized linear mixed-effects models (*n* = 97). The predicted RIs were contained within previously published values and had narrower credible intervals. Most analytes did not vary significantly with foraging ground (76%, 22/29), body mass (86%, 25/29) or curved carapace length (83%, 24/29). Length and body mass effects were found for eosinophils, heterophil:lymphocyte ratio, alkaline phosphatase, aspartate transaminase and urea. Significant differences between foraging grounds were found for albumin, cholesterol, potassium, total protein, triglycerides, uric acid and calcium:phosphorus ratio. We provide derived RIs for foraging green turtles, which will be helpful in future population health assessments and conservation efforts. Future RI studies on threatened species would benefit from adapting established veterinary and biomedical standards.

## Teaser

Reference intervals are essential for assessing wildlife health. However, these cannot be reliably determined with small sample sizes. We used Bayesian modeling to account for sample size limitations and predicted blood biochemical and haematological reference intervals for green turtles (Chelonia mydas) across two foraging populations in North Queensland, Australia.

## Introduction

Blood analyses are routinely used for conservation, ecology and rehabilitation purposes and can indicate population declines and long-term survival challenges (Seminoff and Shanker, 2008; Hamann *et al.,* 2010; Perrault *et al.,* 2017; Stacy and Innis, 2017; Perrault *et al.,* 2021). Indicator species, such as green turtles (*Chelonia mydas*), help in assessing the threats a particular ecosystem or habitat is facing (Aguirre and Lutz, 2004, De Cáceres *et al.*, 2010). Ecosystem functions and services can be assessed by species morphology, behaviour, demography, physiology, biogeochemical composition and socioeconomic importance (Castro Tavares *et al.*, 2019). Green turtles contribute to ecosystem functioning in foraging grounds and nesting beaches by transporting significant amounts of nutrients from nutrient-rich foraging grounds to nutrient-poor nesting beaches (Bjorndal and Jackson, 2002). Although considered migratory in their early life stages and during breeding seasons, green turtles show strict fidelity to foraging grounds as small as 2 km^2^ (Musick and Limpus, 1997; Shimada, 2015) and can reflect the qualitative status of their local habitat. Seagrass meadows are considered essential carbon storage and sequestration sites, and green turtles maintain nutrient-rich areas and contribute to the biodiversity of seagrass species through grazing and seed dispersal (Duarte *et al.,* 2010, Fourqurean *et al.,* 2012, Scott *et al.*, 2020).

A comprehensive understanding of a species’ baseline information is required for correctly interpreting haematological and biochemical data and includes assessing physiology and anatomy, reproductive biology, behaviour, food habits and nutritional requirements, home range and expected parasite fauna (Ryser-Degiorgis, 2013). Interpretation of health status is also dependent on the comparison of blood analyte values with suitable reference intervals (RIs). The utility of RIs for each analyte relies on methodological, physiological and environmental factors. General guidelines for the development of RIs in healthy animals are available for species commonly encountered in the veterinary profession (Friedrichs *et al.,* 2012), and a recent systematic analysis is available for nondomestic species (Moore *et al.,* 2020). These guidelines recommend as selection criteria to include a sufficiently large sample size, typically 100–200 individuals for normally distributed data, and to provide statistical reliability when using frequentist statistics (Jennen-Steinmetz and Wellek, 2005; Geffré *et al.,* 2009; Friedrichs *et al.,* 2012; Wellek *et al.,* 2014; Klee *et al.,* 2018; Moore *et al.,* 2020). Specific guidelines for establishing RIs in sea turtles are also available (Page-Karjian and Perrault, 2020); however, some aspects (e.g. the recommended sample size) differ from standards approved for human or veterinary medicine, such as those of the Clinical Laboratory Standards Institute (CLSI) guidelines or the American Society of Veterinary Clinical Pathology Guidelines (CLSI, 2010; Friedrichs *et al.,* 2012; Wilkinson *et al.,* 2016).

Frequentist statistical methods, which are traditionally used in wildlife research, are best suited to large sample sizes (*n* > 120) and low variability between samples (Friedrichs *et al.,* 2012; Moore *et al.,* 2020). By nature, threatened species research is limited by small sample sizes due to population size and distribution, species biology and behaviour, restricted funding and resources, permitting limitations or short project timeframes (Steidl *et al*., 1997, Bissonette, 1999, Lloyd-Smith *et al.,* 2005, Kéry, 2010, Harden *et al*., 2018, Kophamel *et al.,* 2022). Only a limited number of studies on vertebrate wildlife have collected samples from >120 individuals (see reviews on the topic by Cray, 2015, Moore *et al.,* 2020, Kophamel *et al.,* 2022), which is also true for green turtle biochemical and haematological studies (Supplementary Table S1). In addition, blood values in reptiles may be highly variable across species, populations, sex and life stages, and this has been related to the lack of robust RIs (Stahl, 2006; Mitchell and Tully, 2008; Stacy and Innis, 2017). Following guidelines for developing RIs in threatened species is therefore problematic, and unreliable RIs may lead to false interpretations on population health. This may directly hinder conservation, management and rehabilitation efforts by, for example, overseeing abnormal findings within a population that may lead to false-negative diagnoses and inadequate enforcement measures (Deem and Harris, 2017; Sacchi *et al.,* 2020). As a result, an unnoticed decline in population health will reveal a decline in reproductive output and/or population viability, and will lead to increased caseloads in rehabilitation centres (Hamann *et al.,* 2010; Commonwealth of Australia, 2017; Deem and Harris, 2017). Statistical approaches that account for small sample sizes would therefore provide an increased reliability and clinical utility in the determination of RIs (Steidl *et al*., 1997, Harden *et al*., 2018, Sacchi *et al.,* 2020).

**Figure 1:**
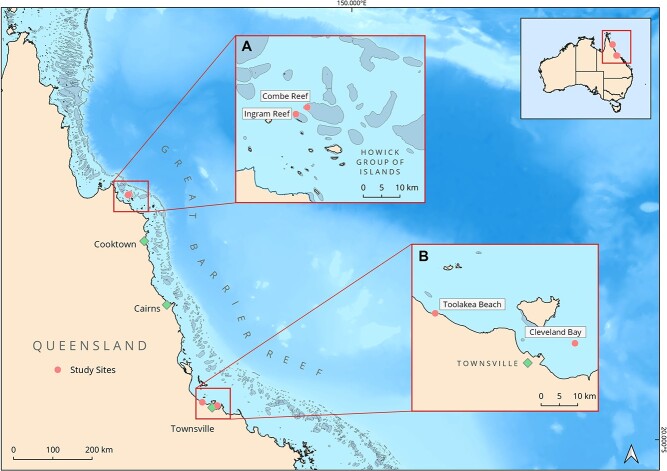
Locations (pink) of the two examined green turtle (*C. mydas*) foraging grounds in North Queensland, Australia. Blood samples were obtained from turtles captured at the offshore Howick Group of Islands location (inset **A**): Combe Reef and Ingram Island; and at the industrialized Townsville region (inset **B**): Cleveland Bay and Toolakea Beach.

While comprehensive and representative sampling and prioritizing large sample sizes remain important considerations, alternative statistical approaches such as Bayesian statistics effectively account for small sample sizes (van de Schoot *et al.,* 2021). Bayesian statistics are very popular in the biomedical and ecological sciences, as they update the probability for a hypothesis as more data becomes available (van de Schoot *et al.,* 2021). Bayesian models are based on the available data and account for individual variation in the calculation of predictive credible intervals, which are equivalent to frequentist 95% confidence intervals (CIs; i.e. mean ± two standard deviations) (Hespanhol *et al*., 2019). As a result, Bayesian models can be used to minimize the number of individuals included in a study (Katki *et al*., 2005, Sottas *et al*., 2011). These characteristics make Bayesian statistics highly suitable for threatened species research.

The aims of this study were to measure biochemical and haematological analytes of green turtle foraging aggregations (*C. mydas, n* = 97), to calculate RIs that could be used for the examined regions and to compare the predicted intervals against other studies. The turtles were sourced from two geographically and ecologically distinct foraging grounds in North Queensland, Australia (Howick Group of Islands and Townsville region; Fig. 1). The examined foraging grounds were in a marine-protected area (MPA) with very little anthropogenic impacts (Howick Group of Islands) and in an MPA located in an industrialized region currently experiencing a port expansion (Townsville region) (Bell *et al*., 2019, Queensland Government, 2021). Green turtle grazing has been reported at both sites (Bell *et al*., 2019, Flint *et al*., 2019). Howick Group of Islands is found in the northern Great Barrier Reef Marine Park (−14.416695°S 144.880484°E), ~30 km from the Cape York region catchment and consists of mid-shelf, unpopulated reefs. The area is considered to be free from chemical pollutants, fishing pressure and coastal development (Villa *et al.,* 2017, Flint *et al*., 2019). In contrast, Townsville region has an estimated population of >230 000 (Australian Bureau of Statistics, 2022) and is influenced by anthropogenic impacts such as industrial runoff, urbanization and coastal dredging (Villa *et al.,* 2017). Based on past studies and the threatened status of this species, we anticipated sampling limitations, and were interested in using statistical methods suitable for small sample sizes (*n* < 120). We aimed to develop Bayesian linear mixed-effects models that would account for the effects of low sample size, geographical location, length and mass on the selected analytes. Further objectives of this study were to compare the resulting intervals in wild turtles between industrialized versus offshore foraging grounds.

## Materials and methods

### Study sites

This study was conducted in two major foraging grounds in North Queensland, Australia: (i) Cleveland Bay (19ᴼ13′05′′S, 146ᴼ55′19″E) and Toolakea Beach (19ᴼ08′40″S, 146ᴼ34′40″E), representing the industrialized Townsville region; and (ii) Combe Reef (14ᴼ25′48″S, 144ᴼ54′42″E) and Ingram Reef (14ᴼ25′03″S, 144ᴼ52′46″E), representing the Howick Group of Islands (14ᴼ30′11″S, 144ᴼ58′26″E) and located offshore (Fig. 1, Table 1). These two major foraging grounds are separated by over 500 km. Sampling was conducted exclusively in winter (between June and August 2019) to avoid travelling during the cyclone season.

**Table 1:** Site locations and timing of blood sampling events of green turtles (*C. mydas*, *n* = 121)

Site	*n*	Date
Townsville region	40	18 June–27 October 2019
Howick Group of Islands	57	9–17 August 2019

(*n*) Number of turtles sampled.

Blood samples were obtained from wild turtles captured in North Queensland, Australia (Townsville region and Howick Group of Islands).

### Animals and sampling protocol

Haematological and biochemical analyte values were determined from plasma obtained from wild turtles (*n* = 97) captured from a boat using rodeo technique (*n* = 85) as described in Limpus and Reed (1985) or hand-captured in shallow water (*n* = 12). Sampling was opportunistic and tide-dependent and predominantly took place in the mornings. A general health assessment was conducted by performing a physical examination to record any injuries, epibiota or presence of tumoral lesions (e.g. fibropapillomatosis) following standard procedures outlined in Deem and Harris (2017) and Harris *et al.* (2017). Only assessed healthy turtles, without macroscopic anomalies, were selected for further examination. Where possible, the animal’s eyes were covered with a cloth to minimize stress. Turtles were tagged with approved titanium identification tags and curved carapace length (CCL) from notch to tip to the nearest 2 mm was measured. Turtles were allocated into life stages based on CCL as per Chaloupka and Limpus (2001) with juveniles (immature) CCL < 65 cm, sub-adults (immature) 65 cm > CCL < 90 cm, and adults (mature) CCL > 90 cm. Body temperature was measured using a thermocouple (8402-20 Thermistor 237 Thermometer, Cole-Palmer Instruments, Vernon Hills, IL, USA), and by inserting the probe 5 cm into the cloaca (Flint, 2013; Stacy and Innis, 2017). Total body mass measurements were recorded using a specially designed harness, which secured each animal around the base of each limb. The harness was then attached to a digital scale where the mass was measured to the nearest 0.1 kg while the animal remained suspended. The harness was removed immediately after weighing. Blood samples were taken from all turtles as described below. Once sampling was completed, the turtles were released in the same area they were captured. Randomly selected juvenile turtles (14.4%, *n* = 14/97) were also assessed by laparoscopic examination to determine their sex. This standard procedure was conducted last and was part of a longitudinal monitoring study conducted on a yearly basis (Bell *et al*., 2019). All turtles were tagged, measured and weighed following standard operating procedures (DBCA, 2017, DES, 2018). The protocol related to measurements and health assessments was standardized and occurred in the following order: capture, physical examination, measurements, blood sampling and laparoscopies (on selected animals). All procedures and protocols were approved by the Great Barrier Reef Marine Park Authority (permit number G19/42769.1) and the Department of Environment and Science, Queensland Government (permit numbers SPP18-001167 and PTU18-001419-2).

### Blood sampling and processing

Blood was sampled from the external jugular vein, which is located on the superficial, lateral regions of the neck. Prior to venepuncture, the skin was disinfected using 70% ethanol swabs (Liv-Wipe, Livingstone, Livingstone Int., Mascot NSW, Australia). Blood samples (2 ml) were collected using a 10-ml syringe (Shandong Hapool Medical Technology Co., Heze, China) with a 22-gauge × 1½ inch needle (Terumo, Japan). No expected or unexpected adverse events occurred. Sample quality was assured by immediate visual inspection of each blood sample. Any sample suspected of contamination with lymph fluid was discarded and an additional sample was collected.

**Figure 2:**
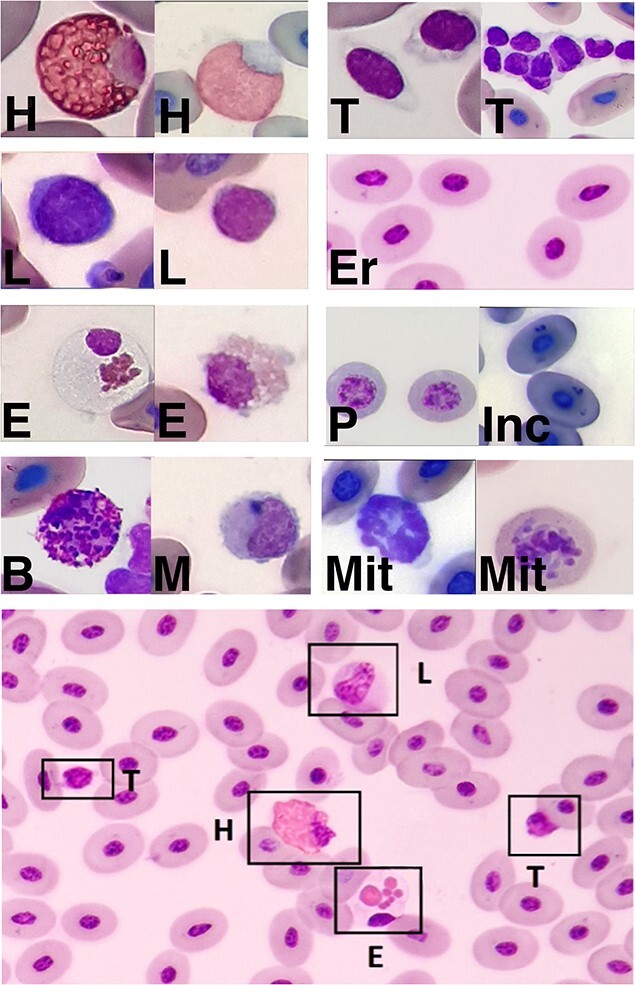
Blood cells of green turtles (*C. mydas*). (H) Heterophil; (L) Lymphocyte; (E) Eosinophil; (B) Basophil; (M) Monocyte; (T) Thrombocytes; (Er) Erythrocytes; (P) Immature erythrocytes; (Inc) Erythrocyte with basophilic inclusions; (Mit) Mitotic figure. Diff Quick and Wright’s stain.

Packed cell volume was determined as an indicator for hydration state and anaemia and was measured twice to determine the average value (Livingstone Microhaematocrit Capillary Tubes, Livingstone Int., Mascot, NSW, Australia; and Pico 17 Microcentrifuge, Thermo Fisher Scientific, Waltham, MA, USA). Duplicate blood smears were prepared using a clean glass slide for the smear and as the spreader slide (Thermo Scientific Menzel-Gläser, Thermo Fisher Scientific, Waltham, MA, USA). The remaining blood was transferred to a sodium-heparin-coated blood collection tube (BD Vacutainer LH 34 I.U., BD Vacutainer Systems, Plymouth, UK), which was gently rocked to ensure proper mixing of blood components. Smears were initially fixated with methanol and were stained once these were returned to the laboratory. Blood smears were interpreted from turtles captured in Townsville region (blood smear quality from turtles captured at Howick Group of Islands was insufficient). Pre-analytical errors may influence analyte values and therefore the RIs determined. Pre- and post-analytical procedures were standardized and followed recommendations for field sampling techniques of reptilian blood (Fullarton, 2012, Eshar *et al.,* 2018). Samples were kept at 4°C (39.2°F), either using refrigeration when available or a cooler box with ice packs for up to 12 hours before centrifugation, and blood tubes were prevented from direct contact with ice packs. Blood smears were stained (Diff Quick and Wright’s stain) and examined using a light microscope (Olympus BX43, Olympus Corp., Tokyo, Japan) at 40× magnification following standard procedures. Blood smears were blindly assessed (JCU Veterinary Diagnostic Pathology Laboratory, Townsville, Queensland, Australia), and leukocyte identification was determined upon consensus (Fig. 2). A white blood cell (i.e. leucocytes) differential count was performed on at least 150 cells, and the cells classified as heterophils, lymphocytes, monocytes, eosinophils or basophils (Wood and Ebanks, 1984, Samour *et al*., 1998). The heterophil:lymphocyte (H:L) ratio was also determined.

Blood samples for biochemical analysis were separated at a maximum relative centrifuge force of 4255 × g for 5 min (Beckman Coulter Allegra X-30R Centrifuge, Brea, CA, USA; samples from Townsville region), or at a maximum relative centrifuge force of 1534 × g for 5 min (E8 Portafuge, LW Scientific, Lawrenceville, GA, USA; samples from Howick Group of Islands). The resulting plasma was frozen at −20°C for up to 2 weeks (Townsville region) or at −80°C (−112°F) for up to 2 months before analysis (Howick Group of Islands) (Kirchgessner and Mitchell, 2009; Marschang, 2014). Plasma samples were thawed and analysed using an automated biochemistry analyser (Beckman Coulter, AU480, Brea, CA, USA), which was regularly used to examine plasma from sea turtles and other wildlife. The clinical biochemists were blinded to the allocation of individual samples to groups (i.e. Howick Group of Islands, Townsville region). Plasma samples with haemolysis scores equal or above two were discarded for packed cell volumes (PCV), total solids and glucose (Stacy and Innis, 2017; Stacy *et al.,* 2019). The lipaemia/turbidity, icterus and hemolysis (LIH) assay did not identify any samples contaminated with lipaemia or icterus. The following analytes were evaluated: albumin (g/l), alkaline phosphatase (U/l), aspartate transaminase (U/l), total bilirubin (μmol/l), calcium (mmol/l), chloride (mmol/l), cholesterol (mmol/l), creatine kinase (U/l), creatinine (μmol/l), globulins (g/l), glucose (mmol/l), lactate dehydrogenase (U/l), magnesium (mmol/l), phosphorus (mmol/l), potassium (mmol/l), total protein (g/l), sodium (mmol/l), triglycerides (mmol/l), urea (mmol/l) and uric acid (mmol/l).

### Statistical analyses

All statistical analyses were produced with R statistical software, using the package ggplot2 for data visualization (Hadley, 2016; R Core Team, 2019). The statistical approaches used in our study were based on the methods used by Logan (2020), Emslie *et al.* (2019), Hannan *et al*. (2021), Sacchi *et al.* (2020) and Spinks *et al.* (2021). Distribution of the response variables (i.e. the biochemical and haematological analytes) were either Gaussian or Gamma, and log-transformed models were considered (normality or non-normality results for each analyte are described in Table 3 and were based on the best model fit). Bayesian generalized linear mixed-effects models were developed for all biochemical and haematological variables, except for sodium and chloride, which were assessed using Bayesian generalized additive models (best model fit). The models were fit using uninformative normal priors or with weak informative priors to allow for regularization whenever a more informative prior was required (Korner-Nievergelt *et al.,* 2015). The posterior prior was derived from the prior distribution, and suitability was confirmed with visual posterior checks. Models were run with the No-U-Turn sampler, using three chains and 5000 iterations. The first 1000 iterations were discarded to converge the model to the correct posterior distribution.

Models were fitted separately for each response variable (i.e. biochemical or haematological analytes). The response variables were first explored graphically and were then statistically analysed by fitting the models previously mentioned. We included location, mass, and CCL as fixed effects, and animal ID as a random effect to account for inter-animal variability. Collinearity between mass and CCL in the studied locations is very common, especially in mature turtles (Bell *et al*., 2019). We included both variables into our calculations to account for exceptions where collinearity might not be the case (e.g. young turtles with an increased growth rate, or turtles that vary in body condition for the same CCL) (Eckert *et al*., 1999). In addition, the uninformative and weak informative priors used in Bayesian statistics help reparametrizing the model, accounting for collinearity within the data (Ogle and Barber, 2020). The resulting predictions were then back transformed, when applicable, to obtain the final RIs in their original scale. The predicted values for each parameter are reported as estimated marginal mean (EMM), and as lower and upper highest posterior density credible intervals (HPDCIs) (Table 3), which are analogous to frequentist CIs (Lee, 1989). HPDCI and CI only differ in the way the predicted parameter is treated, i.e. Bayesian HPDCI treats the predicted parameter as a random variable, whereas frequentist CI treats it as a fixed variable.

All models were fit in a Bayesian analytical framework available in the packages rstanarm (Goodrich, 2020), brms (Bürkner, 2017, 2018) and gamm4 (Wood *et al*., 2017). Model assumptions (e.g. linearity and homogeneity of variance) were visually confirmed with diagnostic residual plots, all of which were satisfactory, using the packages coda (Plummer *et al*., 2006), bayesplot (Gabry and Mahr, 2021), ggmcmc (Fernández-i-Marín, 2016) and DHARMa (Hartig, 2020). The final model selection was based on diagnostic residual plots (e.g. DHARMa residual plotting, Hartig, 2019), on the fit of the data to the selected model and on the corrected Akaike Information Criterion for small sample sizes (AICc, Barton and Barton, 2015). Outlier identification and exclusion was performed with residual plotting using the package DHARMa (Hartig, 2019), and negative analyte values were excluded prior to running the models. Sample size estimates using G^*^Power analysis revealed a total sample size of 159 turtles to achieve a Power of 0.8 (effect size 0.25, α 0.05, three groups). However, this estimate relates specifically to frequentist statistical approaches, since Bayesian methods do not assume fixed/known effect sizes. Posterior prior distributions were derived instead, all of which were satisfactory.

Specific contrasts were conducted for comparisons across locations with the package emmeans (α = 0.05) (Lenth, 2016). Posterior probability distributions using the Markov Chain Monte Carlo (MCMC) estimation assessed the effects of location, mass and CCL on the measured analytes (Fernández-i-Marín, 2016). The differences in the parameter intervals were based on 95% Bayesian Uncertainty Intervals (UIs) for modelled higher posterior density (HPD) median effects. Statistical significance (*P* < 0.05) was inferred when the 95% UIs did not overlap. Whenever referring to location differences throughout the manuscript, it should be noted that mass and CCL were accounted for in the specific contrasts.

**Figure 3:**
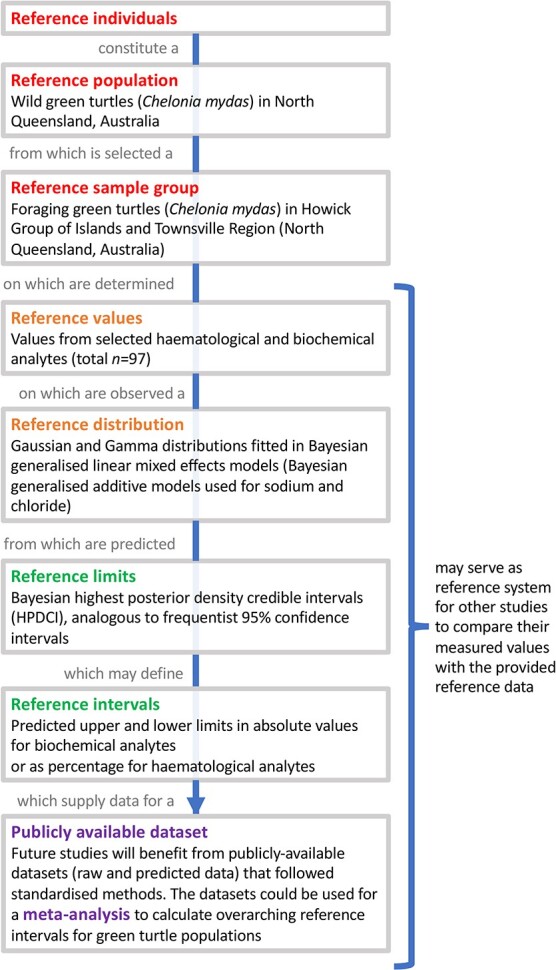
Relationship between the measured analyte values and the calculated RIs according to the CLSI and International Federation of Clinical Chemistry and the Laboratory Medicine document C28-A3.5. Adapted from Higgins (2012).

Correlations between variables were assessed using Pearson’s and Spearman’s correlation coefficient analyses (strong correlation assumed when *P* < 0.05 and *r* > 0.5, Supplementary Appendix S1). Effect size indexes (Hedges’ *g*) were calculated where possible for comparison with other studies (Supplementary Table S3). Additional body condition indices (BCI) were determined by converting straight carapace length from the measured CCL values (Bjorndal and Bolten, 1989; Bjorndal *et al*., 2000; Norton and Wyneken, 2015). This study followed recommended human and veterinary guidelines (Fig. 3) during the data collection and analysis process to ensure the reliability of the established RIs, i.e. CLSI guidelines, FAIR principles, ARRIVE Guidelines (Supplementary Appendix S3) and American Society of Veterinary Clinical Pathology Guidelines (CLSI, 2010, McGrath *et al*., 2010, Friedrichs *et al.,* 2012, Wilkinson *et al.,* 2016, Percie du Sert *et al.,* 2020).

The dataset used for the analyses is available as a spreadsheet saved in MS Excel (.xlsx), Open Document (.ods) and Comma-separated values (.csv) formats in Research Data Australia, at https://doi.org/10.25903/9rm7-k267 (doi: 10.25903/9rm7-k267; Kophamel and Munns, 2022).

## Results

### Animal characteristics

A total of 97 wild turtles were captured. Of these, 26% were adults, 8% subadults and 66% juveniles (Table 2). Laparoscopic examination of a subset of the sample revealed 12 female and 2 male juvenile turtles (*n* = 14/97). The different distribution of life stages across the two sites was reflected in sample distribution, with adults (*n* = 25) and subadults (*n* = 8) only caught at Howick Group of Islands, and the Townsville region group consisting entirely of juveniles (*n* = 40). All turtles appeared healthy on physical examination, were in good body condition and had no apparent external lesions, except from one animal which was missing a front limb (ID number QA94686). Mean CCL of all turtles was 65.5 cm (range, 36.8–115.2 cm), and mean mass was 43.4 kg (range, 6.15–147.2 kg). Mean BCI was 1.16 (range, 0.88–1.53). No significant differences in animal characteristics (i.e. CCL, mass, BCI and cloacal temperature) between sites were identified (Table 2). Mass and CCL were strongly and positively correlated (Pearson’s correlation coefficient 0.97, *P* < 0.05, *t* = 43, *df* = 103). Animal data for each study site and life stage are provided in Table 2.

**Table 2:** Characteristics of the examined wild green turtles (*C. mydas*) organized by sampling locations (mean ± standard deviation)

Life stage and parameter	Townsville region (*n* = 40)	Howick Group of Islands (*n* = 57)	Townsville vs Howick *P*
**Juvenile** (<65 cm CCL) Sample size CCL (mean cm ± SD) Mass (mean kg ± SD) BCI Sex (*n*)^*^ Cloacal temperature (mean °C ± SD) **Subadult** (65–90 cm CCL) Sample size CCL (mean cm ± SD) Mass (mean kg ± SD) BCI Sex (*n*)^*^ Cloacal temperature (mean °C ± SD) **Adult** (>90 cm CCL) Sample size CCL (mean cm ± SD) Mass (mean kg ± SD) BCI Sex (*n*)^*^ Cloacal temperature (mean °C ± SD)	40 46.3 ± 4.8 10.8 ± 3.7 1.2 ± 0.1 U:40 27.7 ± 3.7 NA NA	24 55.0 ± 8.3 20.1 ± 8.3 1.2 ± 0.1 F:12, M:2, U:10 29.3 ± 2.8 8 80.4 ± 6.0 57.3 ± 11.7 1.1 ± 0.0 U:8 31.0 ± 3.6 25 100.6 ± 8.2 111.5 ± 24.1 1.1 ± 0.1 M:4, F:21 30.7 ± 1.8	ns ns ns ns NA NA

^*^(U) undetermined, (F) female, (M) male, (*n*) Number of turtles sampled.

Results for tests of significance (*P*) are displayed for the effects of location (i.e. Howick Group of Islands versus Townsville region) on animal characteristics. (ns) non-significant; (sig) significant. RIs were determined from turtles captured at Townsville region and Howick Group of Islands (*n* = 97).

### RIs for blood biochemical and haematological parameters

RIs (EMM, 95% upper and lower HPDCI limits) are reported in Table 3 (measured, original data are reported in Supplementary Table S2). The majority of blood analytes were not statistically different in the turtles across locations, mass or CCL (76%, 86% and 83%, respectively) (Table 3).

Location was associated with significant (*P* < 0.05) differences in 7/29 (24%) blood analytes: albumin, cholesterol, potassium, total protein, triglycerides, uric acid and calcium:phosphorus ratio (Table 3). Mass and CCL were associated with eosinophil percentage and H:L ratio (*P* < 0.05). Plasma levels of alkaline phosphatase and urea were also influenced by CCL and mass (Table 3). An exception was that CCL had a significant effect on aspartate transaminase (*P* < 0.05), whereas mass did not. These findings support our inclusion of both CCL and mass (correlated variables) in the linear mixed-effects models, as CCL and mass had different effects on analytes. No other analytes were significantly influenced by mass or CCL.

**Table 3:** Haematological (A) and biochemical (B) RIs for wild (*n* = 97) green turtles (*C. mydas*)

(A) Haematological reference intervals for wild turtles captured in Townsville region (*n* = 28)								
Analyte (unit)	Location (*n*)	Distribution	EMM	95% HPDCI		Tests of significance (*P*)		
				Lower limit	Upper limit	Location	Mass	Length
Packed cell volume (%)	Townsville (24)	P	25.1	21.2	29	ns	ns	ns
Heterophils (%)	Townsville (28)	P	45.4	40	50.8	ns	ns	ns
Lymphocytes (%)	Townsville (28)	P	44.8	39.4	50	ns	ns	ns
Monocytes (%)	Townsville (26)	NP	4.1	2.8	6.3	ns	ns	ns
Eosinophils (%)	Townsville (27)	P	4.7	3.3	6.1	ns	**sig**	**sig**
Basophils (%)	Townsville (28)	NP	0.06	0	0.2	ns	ns	ns
Heterophil:lymphocyte ratio (ratio)	Townsville (28)	NP	1.1	0.8	1.4	ns	**sig**	**sig**
(B) Biochemical reference intervals for wild turtles (*n* = 97), separated by location (Townsville region and Howick Group of Islands)								
Analyte (unit)	Location (*n*)	Distribution	EMM	95% HPDCI		Tests of significance (*P*)		
				Lower limit	Upper limit	Location	Mass	CCL
Albumin (g/l)	Townsville (40)	NP	9.3	8.3	10.3	**sig**	ns	ns
	Howick (57)		13.1	11.4	14.8			
Alkaline phosphatase (U/l)	Townsville (40)	NP	15.3	11.9	18.8	ns	**sig**	**sig**
	Howick (57)		18.8	15.2	22.3			
Aspartate transaminase (U/l)	Townsville (37)	NP	190	171	211	ns	ns	**sig**
	Howick (56)		222	197	251			
Total bilirubin (μmol/l)	Townsville (37)	NP	1.9	1.6	2.1	ns	ns	ns
	Howick (56)		1.9	1.7	2.3			
Calcium (mmol/l)	Townsville (40)	NP	1.8	1.6	2.0	ns	ns	ns
	Howick (37)		2.1	1.9	2.4			
Chloride (mmol/l)	Townsville (37)	NP	113	111	115	ns	ns	ns
	Howick (57)		112	111	114			
Cholesterol (mmol/l)	Townsville (39)	P	2.4	2.0	2.8	**sig**	ns	ns
	Howick (55)		4.0	3.5	4.4			
Creatine kinase (U/l)	Townsville (38)	NP	1036.9	814.3	1287.8	ns	ns	ns
	Howick (56)		1363.1	1060.2	1714.9			
Creatinine (μmol/l)	Townsville (21)	NP	4.3	3.1	5.8	ns	ns	ns
	Howick (47)		4.6	3.7	5.7			
Globulins (g/l)	Townsville (40)	NP	22.1	15.2	30.6	ns	ns	ns
	Howick (57)		28.0	20.2	36.7			
Glucose (mmol/l)	Townsville (57)	NP	5.6	5.1	6.1	ns	ns	ns
	Howick (40)		5.0	4.5	5.5			
Lactate dehydrogenase (U/l)	Townsville (57)	NP	205	120	305	ns	ns	ns
	Howick (38)		216	142	301			
Magnesium (mmol/l)	Townsville (57)	NP	4.1	3.1	5.3	ns	ns	ns
	Howick (40)		4.6	3.6	5.8			
Phosphorus (mmol/l)	Townsville (40)	NP	2.1	1.3	3.0	ns	ns	ns
	Howick (57)		1.7	1.1	2.2			
Potassium (mmol/l)	Townsville (40)	P	4.1	3.8	4.4	**sig**	ns	ns
	Howick (57)		4.6	4.3	4.8			
Total protein (g/l)	Townsville (40)	NP	33.9	22.9	46.2	**sig**	ns	ns
	Howick (57)		43.1	31.6	56.7			
Sodium (mmol/l)	Townsville (33)	NP	153	151	154	ns	ns	ns
	Howick (57)		153	152	155			
Triglycerides (mmol/l)	Townsville (39)	NP	0.6	0.3	1.2	**sig**	ns	ns
	Howick (55)		1.0	0.5	1.8			
Urea (mmol/l)	Townsville (36)	NP	3.7	2.3	5.3	ns	**sig**	**sig**
	Howick (57)		4.6	3.1	6.6			
Uric acid (mmol/l)	Townsville (39)	NP	0.05	0.03	0.08	**sig**	ns	ns
	Howick (55)		0.09	0.05	0.13			
Ca:P ratio	Townsville (40)	NP	0.9	0.5	1.4	**sig**	ns	ns
	Howick (57)		1.3	0.8	2.0			
Albumin:globulin ratio	Townsville (40)	NP	0.51	0.36	0.70	ns	ns	ns
	Howick (57)		0.51	0.38	0.66			

Results for tests of significance (*P*) are displayed for the effects of location (i.e. Howick Group of Islands versus Townsville region), mass and CCL. Analyte, unit, sample size (*n*), distribution (P: parametric, NP: non-parametric), EMM, % higher posterior density credible intervals (HPDCI) lower and upper limits (analogous to mean +/− 2 SD) and results of the tests of significance are reported. The differences in the parameter ranges were based on 95% Bayesian UIs for modelled HPDCI. Statistical significance was assessed with posterior probability distributions using the MCMC estimation. Statistical significance (sig) was inferred when the 95% UIs did not overlap (*P* < 0.05).

## Discussion

We present haematological and biochemical blood analyte RIs for two green turtle (*C. mydas*) foraging grounds in North Queensland, Australia (*n* = 97), which were derived using Bayesian predictive modelling. The Bayesian generalized linear mixed-effects models accounted for the effects of low sample size (*n* < 120), geographical location, length and mass. RIs for wild turtles were predicted by including both locations into the Bayesian model, and by accounting for potential differences across them (Table 3).

Our predicted intervals were narrower and within previously reported values or intervals that had been calculated using frequentist statistics (Aguirre and Balazs, 2000, Hamann *et al*., 2006, Whiting *et al*., 2007, Arthur *et al*., 2008, Flint *et al.,* 2010) (Supplementary Table S1). A wide range of factors are known to affect blood analytes in sea turtles, including geographical location, diet composition, sex, age of maturity (i.e. mass and length), captivity, season or weather conditions and sample handling and processing (Herbst and Jacobson, 2002, Hamann *et al*., 2006, Drake *et al.,* 2017, Stacy and Innis, 2017, Harden *et al*., 2018, Sacchi *et al.,* 2020). In our study, most haematological and biochemical analytes had no significant association (*P* > 0.05) with location, mass or CCL (76%, 86% and 83%, respectively), with some exceptions detailed below (Table 3). Other studies, most of which predominantly sampled immature green turtles as well (31/37 studies, 84%, Supplementary Table S1), reported significant effects of CCL and/or mass on the measured blood analytes (Bolten and Bjorndal, 1992, Hasbun *et al.*, 1998, Labrada-Martagon *et al.*, 2010). The difference in the impact of mass and CCL on blood analytes between the current and previous studies may be related to differences in the statistical treatment of our data, location, seasonality and/or diet (Stacy and Innis, 2017).

### Statistical approach

Clinical guidelines recommend establishing RIs with a large enough sample size (*n* > 120) and using predictive statistical models (e.g. linear mixed effects models or Bayesian statistics) to minimize variability within and between analytes (Katki *et al*., 2005, CLSI, 2010, Sottas *et al*., 2011, Friedrichs *et al.,* 2012, Ozarda, 2016, Harden *et al*., 2018, Sacchi *et al.,* 2020, Kophamel *et al.,* 2022). Most veterinary studies refer to the American Society of Veterinary Clinical Pathology (ASVCP) Guidelines (Friedrichs *et al.,* 2012), which have also been promoted by sea turtle researchers (Page-Karjian *et al.,* 2015; Stacy and Innis, 2017; Stacy *et al.,* 2019; de Mello and Alvarez, 2020; Page-Karjian *et al.,* 2020). Other recommendations include sampling a minimum of 20 animals to establish RIs, with larger sample sizes preferred to calculate more reliable results (Page-Karjian and Perrault, 2020). From a clinical perspective, however, Bayesian models have the advantage of accounting for small sample sizes and overcome important limitations of frequentist likelihood models, such as biassed maximum likelihood estimates (Katki *et al*., 2005, van de Schoot *et al.,* 2021). Bayesian statistics also have the ability to incorporate independent information about both fixed and random factors and to fit models when complex and multiple interactions exist between variables (van de Schoot *et al.,* 2021). For example, Bayesian models have been used to establish haematological RIs in lizards (Sacchi *et al.,* 2020), identify abnormal biochemical analytes in veterinary medicine (Knox *et al*., 1998) and predict wildlife population declines over time (King *et al*., 2009). We present our Bayesian modelling as an example for establishing robust RIs in green turtle studies limited by small sample sizes.

A paradigm shift to develop standardized procedures for sea turtles specifically, and for threatened species research in general, has been called out by several authors and organizations (Lawson *et al.*, 2021; Stacy and Innis, 2017; Mashkour *et al.*, 2020; Page-Karjian *et al.,* 2020, Ryser-Degiorgis, 2013, Stokes *et al.*, 2010). Failure to achieve this strategic priority will result in increased false-positive and false-negative diagnoses and unreliable population health estimates. Ultimately, evidence-informed rehabilitation and conservation efforts will be enhanced by accurate and representative RIs. Threatened species studies would therefore benefit from adapting established veterinary and biomedical standards, such as the ASVCP Guidelines (Friedrichs *et al.,* 2012). However, if the recommended sample size (*n* > 120) cannot be reached, using alternative predictive approaches such as Bayesian statistics is strongly encouraged. Previous studies using Bayesian modelling frameworks used or recommended sample sizes ranging from 20 for RIs predictions in box turtles (*Terrapene ornata*) (Harden *et al*., 2018) and 36 for estimating mortality rates in alligator snapping turtles (*Macrochelys temminckii*) (Steen and Robinson Jr, 2017) to 100–140 for sex ratio predictions in loggerhead turtles (*Caretta caretta*) (Shertzer *et al.*, 2018). The minimum sample size to be used with Bayesian models for the determination of RIs should be confirmed with prior predictive checking, which is particularly relevant in complex models with small sample sizes (van de Schoot *et al.,* 2021). If the summary statistics are not satisfactory, alternative distributions, priors, model estimation or increasing the sample size should be attempted. The efficiency of the algorithm can be further assessed by obtaining the effective sample size of the sampled parameter values (van de Schoot *et al.,* 2021). Nevertheless, RI studies should always be carefully designed and aimed for collecting randomized data from as many individuals as possible. Sampling bias might still occur in Bayesian statistics, and the sampled individuals might not accurately represent the population if the sample size is too small. For further information and recommendations on Bayesian modelling, prior selection and sample size, we refer the interested reader to a recent review by van de Schoot *et al.* (2021) and to a comprehensive RI study on lizards (Sacchi *et al.,* 2020).

### Haematological analyses: White blood cell differential counts

Reptilian leukocytes are considered indicators for systemic stressors, with heterophils fulfilling the surrogate role of neutrophils in lower vertebrates (Campbell, 2006, Campbell, 2015, Stacy and Innis, 2017, Flint *et al*., 2019). Heterophils seem to have similar functions to those found in avian blood, as they rely on oxygen-independent mechanisms to combat microorganisms (Stacy *et al*., 2011, Campbell, 2015). We compared our leukocyte percentages (%) with those previously reported for green turtles (Samour *et al*., 1998, Flint *et al.,* 2010, Lewbart *et al.,* 2014, March *et al.,* 2018) and calculated effect size indexes (Hedges’ *g*) and % difference in mean values where possible (Supplementary Table S3). The H:L ratio is a reliable method to estimate stress responses in vertebrates (Davis *et al*., 2008, Krams *et al.,* 2012). Elevated H:L ratios may reflect physiological differences between green turtle aggregations, or indicate a sub-clinical, inflammatory response (Davis *et al*., 2008, Goessling *et al*., 2015). Globally, green turtles are reported to have a low H:L ratio (Lewbart *et al.,* 2014; Muñoz-Pérez *et al.,* 2017); but the opposite trend has also been found in the United Arab Emirates and Australia (Samour *et al*., 1998, March *et al.,* 2018). High H:L ratio has also been found in loggerhead turtles (*Caretta caretta*) in the Atlantic Ocean (Casal *et al*., 2009, Deem *et al.,* 2009, Kelly *et al.,* 2015). The H:L ratio was approximately 1:1 in our turtles. Only two Australian studies to date reported white blood cell counts, both in locations >1000 km South of our field sites; Flint *et al.* (2010) reported a 1:3 H:L ratio for Southern Queensland turtles (Australia) and March *et al.* (2018) reported 4:1 and 2:1 ratios for rehabilitating green turtles in New South Wales (Australia). In our study, mass was found to have a negative effect on the H:L ratio and a positive effect on the eosinophil percentages (%) (Table 3). The various relationships observed suggests age-related changes and increasing exposure to environmental stressors or infectious agents to influence H:L ratios (Aguirre *et al*., 1995, Deem *et al.,* 2009, Oh and Hustead, 2011, Muñoz *et al.,* 2013). This finding is also observed in humans who may experience a dominance shift of lymphocytes to neutrophils with ageing (Li *et al.,* 2015). The increasing eosinophilia in the turtles may reflect a decrease in heterophils, or an increasing parasite burden as the turtles age (Aguirre *et al*., 1995, Deem *et al.,* 2009, Muñoz *et al.,* 2013). Eosinophil percentages (%) of the sampled turtles were lower than those previously reported (Samour *et al.*, 1998, Lewbart *et al.,* 2014). PCV of the turtles included in our study were within previously reported intervals for green turtles (Flint *et al.,* 2010; Lewbart *et al.,* 2014).

### Biochemical analytes

The established biochemical intervals for wild turtles fell within previously reported blood values, ranges or intervals for green turtles in Australian waters and elsewhere (Bolten and Bjorndal, 1992, Aguirre and Balazs, 2000, Hamann *et al*., 2006, Whiting *et al*., 2007, Arthur *et al*., 2008, Flint *et al.,* 2010, March *et al.,* 2018), with some exceptions (effect size and mean % difference are outlined in Supplementary Table S3). Our RIs were narrower than those previously defined for the same regions (Flint *et al.,* 2010), which we attributed to the differences in the statistical methodology used. In this section, we decided to focus on the analytes that differed across studies and refer the interested reader to Stacy and Innis (2017) for a detailed summary on clinical pathology in sea turtles.

*Glucose*—Sick animals often present hypoglycaemia or hyperglycaemia, which is usually associated with a stress response (Innis *et al.,* 2009; Stacy and Innis, 2017). Hypoglycaemia has been associated with exhaustion and prolonged fasting (Deem, 2009, Stacy and Innis, 2017). We found higher plasma glucose levels than reported by Hamann *et al*. (2006), which we associated to methodological differences in the assays used, since the turtles in both studies were deemed to be healthy. Our study utilized a glucose hexokinase method, which on average, has fewer known interferences than the more commonly used glucose oxidase methods (Link *et al.,* 2015, Dickson *et al*., 2019). Interferents with the glucose oxidase method could also reflect the rapidity with which the plasma was separated from the red blood cells (Kunze *et al.,* 2020).

*Enzymes*—Aspartate transaminase was significantly associated with CCL, which suggests age-related changes, i.e. growth (Oh and Hustead, 2011). Alkaline phosphatase, which was influenced by mass and CCL, is an enzyme related to bone formation and osteoblast activity (van Straalen *et al*., 1991). This enzyme has been shown to be higher in juvenile and subadult turtles (Bolten and Bjorndal, 1992), which could have been the case in our study. Our turtles had lower plasma creatine kinase levels than those reported by March *et al.* (2018) in rehabilitating turtles. Elevated creatine kinase could be related to muscle catabolism (e.g. cachectic animals), capture methods and acute stress responses; however, further research is needed to confirm these hypotheses in reptiles (Anderson *et al*., 2013, Petrosky *et al*., 2015).

*Nitrogenous compounds—*The results from our study demonstrated higher urea values than those reported previously (Hamann *et al*., 2006, Whiting *et al*., 2007), which may be related to a higher-protein diet (Singer, 2003, Whiting et al., 2007). Creatinine concentrations measured in this study were also lower than those previously reported (Bolten and Bjorndal, 1992, Aguirre and Balazs, 2000, Hamann *et al*., 2006, Whiting *et al*., 2007, Flint *et al.,* 2010). Analytical differences cannot be excluded either, as creatinine levels in this study were analysed using Jaffe-based chemistry (Supplementary Appendix S4), whereas other laboratories may use an enzymatic method (Delanghe and Speeckaert, 2011). However, creatinine is of minimal clinical relevance in sea turtles, and we decided to disregard this finding (Manire *et al.,* 2002; Innis *et al.,* 2009).

*Electrolytes and minerals—*Sick turtles often present elevated electrolytes (usually sodium, potassium, chloride and phosphorus), which has been linked to dehydration, renal disease, hyperaldosterism or salt gland dysfunction (Innis *et al.,* 2009, Keller *et al.*, 2012, Stacy and Innis, 2017). Electrolytes and minerals may be influenced by diet (in particular calcium, magnesium, sodium or phosphorus) or by the reproductive physiology of nesting females (e.g. calcium), and do not necessarily reflect pathological disorders (Raphael, 2003, Stacy and Innis, 2017, Bloodgood *et al.*, 2019). Calcium and magnesium, for example, are associated with skeletal formation, contribute to the activation of other enzymes and can be found in high concentrations in vegetation (Bloodgood *et al*., 2019). The Ca:P ratio is a strong indicator for UVB deficiency, metabolic bone disease and nutritional secondary hyperparathyroidism in captive reptiles (Perrault *et al.,* 2012; Stacy and Innis, 2017). In this study, the examined turtles exhibited a normal Ca:P ratio. Mildly to markedly inverted Ca:P ratios have also been reported in healthy turtles and might be related to life stage, diet or metabolic imbalances (Stringer *et al.*, 2010, Kelly *et al.,* 2015, Stacy and Innis, 2017). The turtles from this study had lower phosphorus levels than reported by Flint *et al.* (2010), higher magnesium levels than reported by Whiting *et al.* (2007) and lower sodium levels than reported by Bolten and Bjorndal (1992) (Supplementary Table S3). Our findings could be attributed to dietary differences, as no concurrent abnormalities or methodological differences in the assays used were found (Supplementary Appendix S4).

### Location effects

Significant differences between Townsville region and Howick Group of Islands (*P* < 0.05) were found for albumin, total protein, potassium, cholesterol, triglycerides, uric acid and Ca:P ratio (Tables 3 and Supplementary Table S2). All values were still within previously reported blood values, ranges or intervals (Bolten and Bjorndal, 1992, Aguirre and Balazs, 2000, Hamann *et al*., 2006, Whiting *et al*., 2007, Arthur *et al*., 2008, Flint *et al.,* 2010, March *et al.,* 2018). Interestingly, neither mass nor CCL affected any of these parameters. We hypothesize that diet composition contributed to the analyte differences across the two sites (Whiting *et al*., 2007, Stacy *et al*., 2018, Bloodgood *et al*., 2019, Perrault *et al.,* 2020, Putillo *et al*., 2020). For example, Whiting *et al*. (2007) found that green turtles that consumed mainly seagrass had higher plasma protein levels than turtles that consumed algae. Total protein levels were higher at Howick Group of Islands than at Townsville region. It is likely that the foraging grounds at Howick Group of Islands are richer in protein sources due to higher food availability and/or nutritional content. Location differences across the same two capture sites were also reported by Flint *et al*. (2019), who assessed the effects of catastrophic weather events on green turtle blood analytes in, 2014–2015 and in, 2017. Unfortunately, Flint *et al*. (2019) did not provide information on the statistical analyses performed, which prevented comparison of statistical methodologies. Further, there appears to be a lack of research detailing seagrass protein content in these foraging grounds. Other factors to consider that influence total protein in sea turtle species are debilitation or malnutrition (Aguirre *et al*., 1995, Deem *et al.,* 2009; Innis *et al.,* 2009; March *et al.,* 2018) or depletion of energy during nesting (Stacy and Innis, 2017; Page-Karjian *et al.,* 2020). None of the examined turtles, however, was deemed to be unhealthy based on physical examination and on the clinical analyses.

With regards to the other analytes, triglycerides and cholesterol were lowest in the Townsville region. Uric acid levels were low in comparison to other studies and were also lowest in the Townsville region. Uric acid tends to be low in healthy sea turtles (Hamann *et al*., 2006, Innis *et al.,* 2009), is likely related to dietary influences (Jones and Seminoff, 2013, Jones *et al.,* 2013, Barajas-Valero *et al*., 2021) and is sometimes found to be increased in unhealthy and/or stranded turtles (Deem *et al.,* 2009, Innis *et al.,* 2009, March *et al.,* 2018). Since neither mass nor CCL influenced triglycerides, cholesterol or uric acid (Table 3), the observed location differences may be related to the nutritional composition of the foraging grounds and prey availability, rather than to dietary shifts across life stages.

### Study limitations

A number of study limitations should be acknowledged. Although by comparison with other studies in green turtles, the present study’s sample size was large, it was still below the recommended threshold considered to be adequate for the generation of RIs when using frequentist statistics (*n* < 120). This limitation was moderated by using statistical procedures (Bayesian methods) that mitigate the weakened statistical power associated with conventional frequentist statistical analysis. Despite the variation in mass and CCL in our study, two-thirds of turtles sampled were juveniles (66%, *n* = 64/97) and one third (34%, 33/97) were subadult and adult turtles (*n* = 8/97 subadult animals of undetermined sex, *n* = 21/97 adult females, and *n* = 4/97 adult males). From our sample, subadult and adult animals were mainly found in Howick Group of Islands and juvenile animals were mainly found in Townsville region (Table 2). To address this imbalance, our models also accounted for the effects of mass and CCL in the predictions.

## Conclusions

Our study provides biochemical and haematological RIs for wild green turtles foraging in North Queensland, Australia, determined using Bayesian statistics that accounted for the effects of small sample sizes. Our estimated RIs fell within existing intervals and had narrower credible intervals. Location, mass and CCL effects were found for 24%, 14% and 17% of analytes, respectively. We recommend that population-specific RIs are produced with predictive statistical approaches that account for small sample sizes and for the effects of geographical location, length and mass; if they are to be used with confidence to evaluate sea turtle health. Randomized and representative sampling of the target population is essential for the determination of RIs. This is particularly important in threatened species research, which is often subject to sample size limitations. Unreliable predictions may result in false-negative or false-positive diagnoses, which can result in inadequate enforcement measures that may threaten population viability. Evidence-based sea turtle conservation and rehabilitation efforts will be enhanced by using accurate and precise RIs.

## Funding

The Department of Environment and Science (Queensland Government) provided in-kind support for conducting the field trip to Howick Group of Islands. Funding for S.K. was provided by James Cook University [International Postgraduate Research Scholarship] and Sea World Research and Rescue Foundation [SWR/6/2019]. Funders had no role in the design, analysis and reporting of the study. The authors declare no conflict of interest.

## Data availability

The data underlying this article are available in Research Data Australia, at https://doi.org/10.25903/9rm7-k267 [doi: 10.25903/9rm7-k267] (Kophamel and Munns, 2022).

## Supplementary Material

suppl_coac043

## References

[ref1] Aguirre AA , Balazs GH (2000) Blood biochemistry values of green turtles, *Chelonia mydas*, with and without fibropapillomatosis. Comp Haematol Int 10: 132–137.

[ref2] Aguirre AA , Balazs GH, Spraker TR, Gross TS (1995) Adrenal and hematological responses to stress in juvenile green turtles (*Chelonia mydas*) with and without fibropapillomas. Physiol Zool 68: 831–854.

[ref3] Aguirre AA , Lutz PL (2004) Marine turtles as sentinels of ecosystem health: is fibropapillomatosis an indicator? Ecohealth 1: 275–283.

[ref4] Anderson ET , Socha VL, Gardner J, Byrd L, Manire CA (2013) Tissue enzyme activities in the loggerhead sea turtle (*Caretta caretta*). J Zoo Wildl Med 44: 62–69. 23505704 10.1638/1042-7260-44.1.62

[ref5] Arthur KE , Limpus CJ, Whittier JM (2008) Baseline blood biochemistry of Australian green turtles (*Chelonia mydas*) and effects of exposure to the toxic cyanobacterium Lyngbya majuscula. Aust J Zool 56: 23–32.

[ref6] Australian Bureau of Statistics (2022) Region summary: Townsville, 2020 Census, https://dbr.abs.gov.au/region.html?lyr=sa4&rgn=318 (last accessed: 5 April 2022).

[ref7] Barajas-Valero S , Rodríguez-Almonacid C, Rojas-Sereno Z, Moreno-Torres C, Matta NE (2021) Hematology, biochemistry reference intervals, and morphological description of peripheral blood cells for a captive population of *Crocodylus intermedius* in Colombia. Front Vet Sci 8: 694354. 34513969 10.3389/fvets.2021.694354 PMC8427611

[ref8] Barton K , Barton MK (2015) Package ‘mumin’, version 1:18.

[ref9] Bissonette JA (1999) Small sample size problems in wildlife ecology: a contingent analytical approach. Sci Total Environ 652: 1040–1050.

[ref9a] Bell IP , Meager J, van de Merwe JP, Hof CAM (2019) Green turtle (*Chelonia mydas*) population demographics at three chemically distinct foraging areas in the northern Great Barrier Reef. Wildl Biol 5: 65–71. 10.1016/j.scitotenv.2018.10.150 30586791

[ref10] Bjorndal KA , Bolten AB (1989) Comparison of straight-line and over-the-curve measurements for growth rates of green turtles, *Chelonia mydas*. Bull Mar Sci 45: 189–192.

[ref11] Bjorndal KA , Bolten AB, Chaloupka MY (2000) Green turtle somatic growth model: evidence for density dependence. Ecol Appl 10: 269–282.

[ref12] Bjorndal KA , Jackson J (2002) Roles of sea turtles in marine ecosystems: reconstructing the past. Biol Sea Turtles 2: 259.

[ref13] Bloodgood JCG , Norton TM, Hoopes LA, Stacy NI, Hernandez SM (2019) Comparison of hematological, plasma biochemical, and nutritional analytes of rehabilitating and apparently healthy free-ranging Atlantic green turtles (*Chelonia mydas*). J Zoo Wildl Med 50: 69–81. 31120664 10.1638/2017-0250

[ref14] Bolten AB , Bjorndal KA (1992) Blood profiles for a wild population of green turtles (*Chelonia mydas*) in the Southern Bahamas: size-specific and sex-specific relationships. J Wildl Dis 28: 407–413. 1512872 10.7589/0090-3558-28.3.407

[ref15] Bürkner P (2017) brms: an R package for Bayesian multilevel models using Stan. J Stat Softw 80: 1–28.

[ref16] Bürkner P (2018) Advanced Bayesian multilevel modeling with the R package brms. The R Journal 10: 395–411.

[ref17] Campbell T (2006) Clinical pathology of reptiles. In Divers SJ, Mader DR, eds. Reptile Medicine and Surgery. Elsevier Health Sciences, pp. 453–470, 10.1016/B0-72-169327-X/50032-8. Saint Louis, MO, USA.

[ref18] Campbell TW (2015) Peripheral blood of reptiles. In Exotic Animal Hematology and Cytology: Campbell/Exotic. John Wiley & Sons, Inc., pp. 67–87, 10.1002/9781118993705.ch3. Hoboken, NJ, USA.

[ref19] Casal AB , Camacho M, López-Jurado LF, Juste C, Orós J (2009) Comparative study of hematologic and plasma biochemical variables in Eastern Atlantic juvenile and adult nesting loggerhead sea turtles (*Caretta caretta*). Vet Clin Pathol 38: 213–218. 19192261 10.1111/j.1939-165X.2008.00106.x

[ref20] Castro Tavares D , Moura JF, Acevedo-Trejos E, Merico A (2019) Traits shared by marine megafauna and their relationships with ecosystem functions and services. Front Mar Sci 6: 262.

[ref21] Chaloupka M , Limpus C (2001) Trends in the abundance of sea turtles resident in southern Great Barrier Reef waters. Biol Conserv 102: 235–249.

[ref22] CLSI (2010) Defining, Establishing and Verifying Reference Intervals in the Clinical Laboratory; Approved Guideline, Ed 3. CLSI document EP28-A3c, Wayne, PA: Clinical and Laboratory Standards Institute.

[ref23] Commonwealth of Australia (2017) Recovery Plan for Marine Turtles in Australia.

[ref24] Cray C (2015) Reference intervals in avian and exotic hematology. Vet Clin 18: 105–116. 10.1016/j.cvex.2014.09.006 25421029

[ref25] Davis A , Maney D, Maerz J (2008) The use of leukocyte profiles to measure stress in vertebrates: a review for ecologists. Funct Ecol 22: 760–772.

[ref26] DBCA Department of Biodiversity Conservation and Attractions (2017) Standard Operating Procedure: Marking of Marine Turtles Using Flipper and PIT Tags. Department of Biodiversity, Conservation and Attractions, Perth, WA.

[ref27] De Cáceres M , Legendre P, Moretti M (2010) Improving indicator species analysis by combining groups of sites. Oikos 119: 1674–1684.

[ref28] Deem SL , Harris HS (2017) Health assessments. In CA Manire, TM Norton, B Stacy, CA Harms, CJ Innis, eds, Sea Turtle Health and Rehabilitation. J Ross Publishing, Plantation, FL, pp. 945–958.

[ref29] Deem SL , Norton TM, Mitchell M, Segars A, Alleman AR, Cray C, Poppenga RH, Dodd M, Karesh WB (2009) Comparison of blood values in foraging, nesting, and stranded loggerhead turtles (*Caretta caretta*) along the coast of Georgia, USA. J Wildl Dis 45: 41–56. 19204334 10.7589/0090-3558-45.1.41

[ref30] Delanghe JR , Speeckaert MM (2011) Creatinine determination according to Jaffe—what does it stand for? NDT Plus 4: 83–86. 25984118 10.1093/ndtplus/sfq211 PMC4421578

[ref31] DES Department of Environment and Science (2018) Marine Turtle Conservation Strategy - Queensland. Department of Environment and Science, Queensland Government, Brisbane.

[ref90] de Mello DMD , Alvarez MCL (2020) Health assessment of juvenile green turtles in southern Sao Paulo State, Brazil: a hematologic approach. J Vet Diagn Invest 32: 25–35. 31845622 10.1177/1040638719891972 PMC7003234

[ref32] Dickson LM , Buchmann EJ, Van Rensburg CJ, Norris SA (2019) The impact of differences in plasma glucose between glucose oxidase and hexokinase methods on estimated gestational diabetes mellitus prevalence. Sci Rep 9: 7238–7237. 31076622 10.1038/s41598-019-43665-x PMC6510785

[ref33] Drake KK , Bowen L, Lewison RL, Esque TC, Nussear KE, Braun J, Waters SC, Miles AK (2017) Coupling gene-based and classic veterinary diagnostics improves interpretation of health and immune function in the Agassiz's desert tortoise (*Gopherus agassizii*). Conserv Physiol 5: 17. 10.1093/conphys/cox037 PMC5550616 28835840

[ref34] Duarte CM , Marbà N, Gacia E, Fourqurean JW, Beggins J, Barrón C, Apostolaki ET (2010) Seagrass community metabolism: assessing the carbon sink capacity of seagrass meadows. Global Biogeochem Cycles 24: GB4032.

[ref35] Eckert KL , Bjorndal KA, Abreu-Grobois FA, Donnelly M (1999) Research and management techniques for the conservation of sea turtles.

[ref36] Emslie MJ , Logan M, Cheal AJ (2019) The distribution of planktivorous damselfishes (*Pomacentridae*) on the Great Barrier Reef and the relative influences of habitat and predation. Diversity 11: 33.

[ref37] Eshar D , Avni-Magen N, Kaufman E, Beaufrère H (2018) Effects of time and storage temperature on selected biochemical analytes in plasma of red-eared sliders (*Trachemys scripta elegans*). Am J Vet Res 79: 852–857. 30058847 10.2460/ajvr.79.8.852

[ref38] Fernández-i-Marín X (2016) ggmcmc: analysis of MCMC samples and Bayesian inference. J Stat Softw 70: 1–20.

[ref39] Flint M (2013) Free-ranging sea turtle health. In J Wyneken, JA Musick, KJ Lohmann, eds, The Biology of Sea Turtles, Vol. 3, pp. 399–417.

[ref40] Flint M , Brand AF, Bell IP, Hof CAM (2019) Monitoring the health of green turtles in northern Queensland post catastrophic events. Sci Total Environ 660: 586–592. 30641386 10.1016/j.scitotenv.2019.01.065

[ref41] Flint M , Morton JM, Limpus CJ, Patterson-Kane JC, Murray PJ, Mills PC (2010) Development and application of biochemical and haematological reference intervals to identify unhealthy green sea turtles (*Chelonia mydas*). Vet J 185: 299–304. 19709912 10.1016/j.tvjl.2009.06.011

[ref42] Fourqurean JW , Duarte CM, Kennedy H, Marbà N, Holmer M, Mateo MA, Apostolaki ET, Kendrick GA, Krause-Jensen D, McGlathery KJ et al. (2012) Seagrass ecosystems as a globally significant carbon stock. Nat Geosci 5: 505–509.

[ref43] Friedrichs KR , Harr KE, Freeman KP, Szladovits B, Walton RM, Barnhart KF, Blanco-Chavez J, American Society for Veterinary Clinical Pathology (2012) ASVCP reference interval guidelines: determination of de novo reference intervals in veterinary species and other related topics. Vet Clin Pathol 41: 441–453. 23240820 10.1111/vcp.12006

[ref44] Fullarton CJ (2012) Pre-analytical errors and their influence on haematological parameters of blood from the green sea turtle, *Chelonia mydas*. MSc Thesis, James Cook University.

[ref45] Gabry J , Mahr T (2021) Bayesplot: plotting for Bayesian models. R package, version 1. https://mc-stan.org/bayesplot/.

[ref46] Geffré A , Friedrichs K, Harr K, Concordet D, Trumel C, Braun J-P (2009) Reference values: a review. Vet Clin Pathol 38: 288–298. 19737162 10.1111/j.1939-165X.2009.00179.x

[ref47] Goessling JM , Kennedy H, Mendonça MT, Wilson AE (2015) A meta-analysis of plasma corticosterone and heterophil: lymphocyte ratios–is there conservation of physiological stress responses over time? Funct Ecol 29: 1189–1196.

[ref48] Goodrich B GJ , Ali I, Brilleman S (2020) rstanarm: Bayesian applied regression modeling via Stan, *R package version* 2:1, https://mc-stan.org/rstanarm.

[ref49] Hadley W (2016) ggplot2: Elegant Graphics for Data Analysis. Springer.

[ref50] Hamann M , Godfrey M, Seminoff J, Arthur K, Barata P, Bjorndal K, Bolten A, Broderick A, Campbell L, Carreras C et al. (2010) Global research priorities for sea turtles: informing management and conservation in the 21st century. Endanger Species Res 11: 245–269.

[ref51] Hamann M , Schäuble CS, Simon T, Evans S (2006) Demographic and health parameters of green sea turtles *Chelonia mydas* foraging in the Gulf of Carpentaria, Australia. Endanger Species Res 2: 81–88.

[ref52] Hannan KD , McMahon SJ, Munday PL, Rummer JL (2021) Contrasting effects of constant and fluctuating pCO2 conditions on the exercise physiology of coral reef fishes. Mar Environ Res 163: 105224. 33316710 10.1016/j.marenvres.2020.105224

[ref53] Harden LA , Fernandez J, Milanovich JR, Struecker BP, Midway SR (2018) Blood biochemical reference intervals for wild ornate box turtles (*Terrapene ornata*) during the active season. J Wildl Dis 54: 587–591. 29561712 10.7589/2017-09-222

[ref54] Harris SH , Flint M, Stewart KM, Harms CA (2017) Field techniques. In CA Manire, TM Norton, B Stacy, CA Harms, CJ Innis, eds, Sea turtle Health and Rehabilitation. J Ross Publishing, Plantation, FL, pp. 819–857.

[ref55] Hartig F (2019) DHARMa: residual diagnostics for hierarchical (multi-level/mixed) regression models. R package version 02: 4.

[ref56] Hartig F (2020) DHARMa: residual diagnostics for hierarchical (multi-level/mixed) regression models. R package version 0330: 4.

[ref57] Hasbun CR , Lawrence AJ, Naldo J, Samour JH, Al-Ghais SM (1998) Normal blood chemistry of free-living green sea turtles, *Chelonia mydas*, from the United Arab Emirates. Comp Haematol Int 8: 174–177.

[ref58] Herbst LH , Jacobson ER (2002) Practical approaches for studying sea turtle health and disease. The Biology of Sea Turtles 2: 385. CRC Press, Boca Raton, FL, USA.

[ref59] Hespanhol L , Vallio CS, Costa LM, Saragiotto BT (2019) Understanding and interpreting confidence and credible intervals around effect estimates. Braz J Phys Ther 23: 290–301. 30638956 10.1016/j.bjpt.2018.12.006 PMC6630113

[ref60] Higgins C (2012) An introduction to reference intervals (1)—some theoretical considerations. Point Care 11: 2–5.

[ref61] Innis CJ , Ravich JB, Tlusty MF, Hoge MS, Wunn DS, Boerner-Neville LB, Merigo C, Weber ES III (2009) Hematologic and plasma biochemical findings in cold-stunned Kemp's ridley turtles: 176 cases (2001–2005). J Am Vet Med Assoc 235: 426–432. 19681727 10.2460/javma.235.4.426

[ref62] Jennen-Steinmetz C , Wellek S (2005) A new approach to sample size calculation for reference interval studies. Stat Med 24: 3199–3212. 16189809 10.1002/sim.2177

[ref63] Jones CE , Stacy NI, Wellehan JFX, Stacy BA, Norton TM, Innis CJ, Nelson S, Keller JM, Arendt M, Segars A et al. (2013) Diagnostic performance of plasma uric acid, magnesium and other biochemical parameters in the diagnosis of renal insufficiency in sea turtles. In Proceedings of the 44th Annual Conference of the International Association for Aquatic Animal Medicine, Sausalito, California, pp. 136–137. Conference was hosted by the International Association for Aquatic Animal Medicine in Sausalito, CA, USA.

[ref64] Jones TT , Seminoff JA (2013) Feeding biology: advances from field-based observations, physiological studies, and molecular techniques. The Biology of Sea Turtles, Vol. III 211–247. CRC Press, Boca Raton, FL, USA.

[ref65] Katki HA , Engels EA, Rosenberg PS (2005) Assessing uncertainty in reference intervals via tolerance intervals: application to a mixed model describing HIV infection. Stat Med 24: 3185–3198. 16189804 10.1002/sim.2171

[ref65a] Keller KA , Innis CJ, Tlusty MF, Kennedy AE, Bean SB, Cavin JM, Merigo C (2012) Metabolic and respiratory derangements associated with death in cold-stunned Kemp's ridley turtles (Lepidochelys kempii): 32 cases (2005–2009). J Am Vet Med Assoc 240: 317–323. 22256849 10.2460/javma.240.3.317

[ref66] Kelly TR , McNeill JB, Avens L, Hall AG, Goshe LR, Hohn AA, Godfrey MH, Mihnovets AN, Cluse WM, Harms CA (2015) Clinical pathology reference intervals for an in-water population of juvenile loggerhead sea turtles (*Caretta caretta*) in Core Sound, North Carolina, USA. PLoS One 10: e0115739. 25738772 10.1371/journal.pone.0115739 PMC4349656

[ref67] Kéry M (2010) Introduction to WinBUGS for Ecologists: Bayesian Approach to Regression, ANOVA, Mixed Models and Related Analyses. Academic Press, 10.1016/B978-0-12-378605-0.00003-X. Cambridge, MA, USA.

[ref68] King R , Morgan B, Gimenez O, Brooks S (2009) Bayesian Analysis for Population Ecology. CRC Press, 10.1201/9781439811887. New York, NY, USA.

[ref69] Kirchgessner M , Mitchell MA (2009) Manual of Exotic Pet Practice. Elsevier, pp. 207–249. 10.1016/B978-141600119-5.50012-3. Saint Louis, MO, USA.

[ref70] Klee GG , Ichihara K, Ozarda Y, Baumann NA, Straseski J, Bryant SC, Wood-Wentz CM (2018) Reference intervals: comparison of calculation methods and evaluation of procedures for merging reference measurements from two US medical centers. Am J Clin Pathol 150: 545–554. 30169553 10.1093/ajcp/aqy082

[ref71] Knox KMG , Reid SWJ, Love S, Murray M, Gettinby G (1998) Application of probability techniques to the objective interpretation of veterinary clinical biochemistry data. Vet Rec 142: 323–327. 9571754 10.1136/vr.142.13.323

[ref72] Kophamel S , Illing B, Ariel E, Difalco M, Skerratt LF, Hamann M, Ward LC, Méndez D, Munns SL (2022) Importance of health assessments for conservation in noncaptive wildlife. Conserv Biol 36: e13724. 33634525 10.1111/cobi.13724 PMC9291856

[ref73] Kophamel S , Munns SL (2022) Data from: Haematological and biochemical reference intervals for wild green turtles (*Chelonia mydas*): a Bayesian approach for small sample sizes. James Cook University. 10.25903/9rm7-k267. PMC10020984 36937701

[ref74] Korner-Nievergelt F , Roth T, Von Felten S, Guélat J, Almasi B, Korner-Nievergelt P (2015) Bayesian Data Analysis in Ecology Using Linear Models with R, Bugs, and Stan. Academic Press, 10.1016/B978-0-12-801370-0.00004-6. Boston, MA, USA.

[ref75] Krams I , Vrublevska J, Cirule D, Kivleniece I, Krama T, Rantala MJ, Sild E, Hõrak P (2012) Heterophil/lymphocyte ratios predict the magnitude of humoral immune response to a novel antigen in great tits (*Parus major*). Comp Biochem Physiol Part A Physiol 161: 422–428. 10.1016/j.cbpa.2011.12.018 22245489

[ref76] Kunze PE , Perrault JR, Chang Y-M, Manire CA, Clark S, Stacy NI (2020) Pre-/analytical factors affecting whole blood and plasma glucose concentrations in loggerhead sea turtles (*Caretta caretta*). PLoS One 15: e0229800. 32126109 10.1371/journal.pone.0229800 PMC7053744

[ref77] Labrada-Martagon V , Mendez-Rodriguez LC, Gardner SC, Lopez-Castro M, Zenteno-Savin T (2010) Health indices of the green turtle (*Chelonia mydas*) along the Pacific Coast of Baja California Sur, Mexico. I. Blood biochemistry values. Chelonian Conserv Biol 9: 162–172.

[ref140] Lawson B , Neimanis A, Lavazza A, López-Olvera JR, Tavernier P, Billinis C, Duff JP, Mladenov DT, Rijks JM and Savić S (2021) How to start up a national wildlife health surveillance programme. Animals 11: 2543. 34573509 10.3390/ani11092543 PMC8467383

[ref78] Lee PM (1989) Bayesian Statistics. Oxford University Press, London.

[ref79] Lenth RV (2016) Least-squares means: the R package lsmeans. J Stat Softw 1: 2016.

[ref80] Lewbart GA , Hirschfeld M, Brothers JR, Muñoz-Pérez JP, Denkinger J, Vinueza L, García J, Lohmann KJ (2014) Blood gases, biochemistry, and hematology of Galapagos green turtles (*Chelonia mydas*). PLoS One 9: e96487. 24824065 10.1371/journal.pone.0096487 PMC4019482

[ref81] Li J , Chen Q, Luo X, Hong J, Pan K, Lin X, Liu X, Zhou L, Wang H, Xu Y et al. (2015) Neutrophil-to-lymphocyte ratio positively correlates to age in healthy population. J Clin Lab Anal 29: 437–443. 25277347 10.1002/jcla.21791 PMC6807196

[ref82] Limpus C , Reed P (1985) The green turtle, *Chelonia mydas*, in Queensland: a preliminary description of the population structure in a coral reef feeding ground. In G Grigg, R Shine, H Ehmann, eds, Biology of Australasian Frogs and Reptiles. Royal Zoological Society of New South Wales, Sydney, Australia, pp. 47–52.

[ref83] Link M , Schmid C, Pleus S, Baumstark A, Rittmeyer D, Haug C, Freckmann G (2015) System accuracy evaluation of four systems for self-monitoring of blood glucose following ISO 15197 using a glucose oxidase and a hexokinase-based comparison method. J Diabetes Sci Technol 9: 1041–1050. 25872967 10.1177/1932296815580161 PMC4667333

[ref84] Lloyd-Smith JO , Cross PC, Briggs CJ, Daugherty M, Getz WM, Latto J, Sanchez MS, Smith AB, Swei A (2005) Should we expect population thresholds for wildlife disease? Trends Ecol Evol 20: 511–519. 16701428 10.1016/j.tree.2005.07.004

[ref85] Logan M (2020) Biostatistical Design and Analysis Using R. Advanced Statistics and Programming Course. Australia, Australian Institute of Marine Science, Queensland.

[ref86] Manire CA , Rhinehart HL, Sutton DA, Thompson EH, Rinaldi MG, Buck JD, Jacobson E (2002) Disseminated mycotic infection caused by *Colletotrichum acutatum* in a Kemp's ridley sea turtle (*Lepidochelys kempi*). J Clin Microbiol 40: 4273–4280. 12409409 10.1128/JCM.40.11.4273-4280.2002 PMC139682

[ref87] March DT , Vinette-Herrin K, Peters A, Ariel E, Blyde D, Hayward D, Christidis L, Kelaher BP (2018) Hematologic and biochemical characteristics of stranded green sea turtles. J Vet Diagn Invest 30: 423–429. 29436286 10.1177/1040638718757819 PMC6505802

[ref88] Marschang RE (2014). Clinical virology. In Current Therapy in Reptile Medicine and Surgery, pp. 32–52. Elsevier Saunders, St. Louis, MO. 10.1016/B978-1-4557-0893-2.00005-3.

[ref141] Mashkour N , Jones K, Kophamel S, Hipolito T, Ahasan S, Walker G, Jakob-Hoff R, Whittaker M, Hamann M, Bell I et al. (2020) Disease risk analysis in sea turtles: A baseline study to inform conservation efforts. PLoS One 15: e0230760. 33095793 10.1371/journal.pone.0230760 PMC7584443

[ref89] McGrath J , Drummond G, McLachlan E, Kilkenny C, Wainwright C (2010) Guidelines for reporting experiments involving animals: the ARRIVE guidelines. Br J Pharmacol 160: 1573–1576. 20649560 10.1111/j.1476-5381.2010.00873.x PMC2936829

[ref91] Mitchell M , Tully TN (2008) Manual of Exotic Pet Practice. Elsevier Health Sciences.

[ref92] Moore AR , Camus MS, Harr K, Kjelgaard-Hansen M, Korchia J, Jeffery U, Paltrinieri S, Pratt SM, Szladovits B (2020) Systematic evaluation of 106 laboratory reference data articles from nondomestic species published from 2014 to 2016: assessing compliance with reference interval guidelines. J Zoo Wildl Med 51: 469–477. 33480521 10.1638/2019-0186

[ref93] Muñoz FA , Estrada-Parra S, Romero-Rojas A, Gonzalez-Ballesteros E, Work TM, Villaseñor-Gaona H, Estrada-Garcia I (2013) Immunological evaluation of captive green sea turtle (*Chelonia mydas*) with ulcerative dermatitis. J Zoo Wildl Med 44: 837–844. 10.1638/2010-0228R4.1. 24450041

[ref94] Muñoz-Pérez JP , Lewbart GA, Hirschfeld M, Alarcón-Ruales D, Denkinger J, Castañeda JG, García J, Lohmann KJ (2017) Blood gases, biochemistry and haematology of Galápagos hawksbill turtles (*Eretmochelys imbricata*). Conservation. Phys Ther 5: cox028. 10.1093/conphys/cox028. PMC5424066 28496982

[ref95] Musick JA , Limpus CJ (1997) Habitat utilization and migration in juvenile sea turtles. In The Biology of Sea Turtles Vol 1, pp. 137–163. CRC Press, Boca Raton, FL, USA.

[ref96] Norton T , Wyneken J (2015) Body Condition Scoring the Sea Turtle. LafeberVet, https://lafeber.com/vet/body-condition-scoring-the-sea-turtle/ (last accessed 5 April 2022).

[ref97] Ogle K , Barber JJ (2020) Ensuring identifiability in hierarchical mixed effects Bayesian models. Ecol Appl 30: e02159. 32365250 10.1002/eap.2159

[ref98] Oh R , Hustead TR (2011) Causes and evaluation of mildly elevated liver transaminase levels. Am Fam Physician 84: 1003–1008. 22046940

[ref99] Ozarda Y (2016) Reference intervals: current status, recent developments and future considerations. Biochem Med 26: 5–16. 10.11613/BM.2016.001 PMC4783089 26981015

[ref100] Page-Karjian A , Chabot R, Stacy NI, Morgan AS, Valverde RA, Stewart S, Coppenrath CM, Manire CA, Herbst LH, Gregory CR et al. (2020) Comprehensive health assessment of green turtles *Chelonia mydas* nesting in southeastern Florida, USA. Endanger Species Res 42: 21–35.

[ref101] Page-Karjian A , Perrault JR (2020) Sea turtle health assessments: maximizing turtle encounters to better understand health. In B Nahill, ed, Sea Turtle Research and Conservation, Lessons from Working in the Field, pp. 31–44. Academic Press, MA, USA.

[ref102] Page-Karjian A , Rivera S, Torres F, Diez C, Moore D, Van Dam R, Brown C (2015) Baseline blood values for healthy free-ranging green sea turtles (*Chelonia mydas*) in Puerto Rico. Comp Clin Pathol 24: 567–573.

[ref103] Percie du Sert N , Ahluwalia A, Alam S, Avey MT, Baker M, Browne WJ, Clark A, Cuthill IC, Dirnagl U, Emerson M et al. (2020) Reporting animal research: explanation and elaboration for the ARRIVE guidelines 2.0. PLoS Biol 18: e3000411. 32663221 10.1371/journal.pbio.3000411 PMC7360025

[ref104] Perrault JR , Arendt MD, Schwenter JA, Byrd JL, Harms CA, Cray C, Tuxbury KA, Wood LD, Stacy NI (2020) Blood analytes of immature Kemp’s ridley sea turtles (*Lepidochelys kempii*) from Georgia, USA: reference intervals and body size correlations. Conserv Physiol 8: coaa091. 33304585 10.1093/conphys/coaa091 PMC7720087

[ref105] Perrault JR , Levin M, Mott CR, Bovery CM, Bresette MJ, Chabot RM, Gregory CR, Guertin JR, Hirsch SE, Ritchie BW et al. (2021) Insights on immune function in free-ranging green sea turtles (*Chelonia mydas*) with and without fibropapillomatosis. Animals 11: 861. 33803547 10.3390/ani11030861 PMC8003005

[ref106] Perrault JR , Miller DL, Eads E, Johnson C, Merrill A, Thompson LJ, Wyneken J (2012) Maternal health status correlates with nest success of leatherback sea turtles (*Dermochelys coriacea*) from Florida. PLoS One 7: e31841. 22359635 10.1371/journal.pone.0031841 PMC3281022

[ref107] Perrault JR , Stacy NI, Lehner AF, Mott CR, Hirsch S, Gorham JC, Buchweitz JP, Bresette MJ, Walsh CJ (2017) Potential effects of brevetoxins and toxic elements on various health variables in Kemp's ridley (*Lepidochelys kempii*) and green (*Chelonia mydas*) sea turtles after a red tide bloom event. Sci Total Environ 605, 606: 967–979. 28693110 10.1016/j.scitotenv.2017.06.149

[ref108] Petrosky KY , Knoll JS, Innis C (2015) Tissue enzyme activities in Kemp's ridley turtles (*Lepidochelys kempii*). J Zoo Wildl Med 46: 637–640. 26352978 10.1638/2015-0014.1

[ref109] Plummer M , Best N, Cowles K, Vines K (2006) CODA: convergence diagnosis and output analysis for MCMC. R News 6: 7–11.

[ref110] Putillo AR , Flint M, Seminoff JA, Spencer RGM, Fuentes M (2020) Plasma biochemistry profiles of juvenile green turtles (*Chelonia mydas*) from the Bahamas with a potential influence of diet. J Wildl Dis 56: 768–780. 33600601 10.7589/JWD-D-20-00009

[ref111] Queensland Government (2021) Townsville Port Expansion Project, State Development I, Local Government and Planning, Project Overview of the Townsville Port Expansion Project.

[ref112] R Core Team (2019) R: A Language and Environment for Statistical Computing. R Foundation for Statistical Computing.

[ref113] Raphael B (2003) Chelonians (turtles, tortoises). Zoo and Wild Animal Medicine. Saunders, St. Louis, Missouri, pp. 48–58.

[ref114] Ryser-Degiorgis MP (2013) Wildlife health investigations: needs, challenges and recommendations. BMC Vet Res 9: 223. 24188616 10.1186/1746-6148-9-223 PMC4228302

[ref115] Sacchi R , Mangiacotti M, Scali S, Coladonato AJ, Pitoni S, Falaschi M, Zuffi MAL (2020) Statistical methodology for the evaluation of leukocyte data in wild reptile populations: a case study with the common wall lizard (*Podarcis muralis*). PLoS One 15: 15. 10.1371/journal.pone.0237992 PMC7449502 32845912

[ref116] Samour JH , Howlett JC, Silvanose C, Hasbun CR, Al-Ghais SM (1998) Normal haematology of free-living green sea turtles (*Chelonia mydas*) from the United Arab Emirates. Comp Haematol Int 8: 102–107.

[ref118] Scott AL , York PH, Rasheed MA (2020) Green turtle (*Chelonia mydas*) grazing plot formation creates structural changes in a multi-species Great Barrier Reef seagrass meadow. Mar Environ Res 162: 105183. 33065522 10.1016/j.marenvres.2020.105183

[ref119] Seminoff JA , Shanker K (2008) Marine turtles and IUCN Red Listing: a review of the process, the pitfalls, and novel assessment approaches. J Exp Mar Biol Ecol 356: 52–68.

[ref120] Shertzer KW , Avens L, Braun McNeill J, Goodman Hall A, Harms CA (2018) Characterizing sex ratios of sea turtle populations: a Bayesian mixture modeling approach applied to juvenile loggerheads (*Caretta caretta*). J Exp Mar Biol Ecol 504: 10–19.

[ref121] Shimada T (2015) Spatial ecology and conservation of sea turtles in coastal foraging habitat. PhD thesis, James Cook University.

[ref122] Singer MA (2003) Dietary protein-induced changes in excretory function: a general animal design feature. Comp Biochem Physiol Part B Biochem Mol Biol 136: 785–801. 10.1016/j.cbpc.2003.08.012 14662303

[ref123] Sottas PE , Kapke GF, Vesterqvist O, Leroux JM (2011) Patient-specific measures of a biomarker for the generation of individual reference intervals: hemoglobin as example. Transl Res 158: 360–368. 22061043 10.1016/j.trsl.2011.08.005

[ref124] Spinks RK , Bonzi LC, Ravasi T, Munday PL, Donelson JM (2021) Sex and time specific parental effects of warming on reproduction and offspring quality in a coral reef fish. Evol Appl 14: 1145–1158. 33897826 10.1111/eva.13187 PMC8061261

[ref125] Stacy N , Innis C (2017) Clinical pathology. In FL Plantation, ed, Sea Turtle Health and Rehabilitation. Plantation, FL, USA: J Ross Publishing, pp. 147–207.

[ref126] Stacy NI , Alleman AR, Sayler KA (2011) Diagnostic hematology of reptiles. Clin Lab Med 31: 87–108. 21295724 10.1016/j.cll.2010.10.006

[ref127] Stacy NI , Bjorndal KA, Perrault JR, Martins HR, Bolten AB (2018) Blood analytes of oceanic-juvenile loggerhead sea turtles (*Caretta caretta*) from Azorean waters: reference intervals, size-relevant correlations and comparisons to neritic loggerheads from Western Atlantic coastal waters. Conservation. Phys Ther 6: coy006. 10.1093/conphys/coy006. PMC5814815 29479433

[ref128] Stacy NI , Chabot RM, Innis CJ, Cray C, Fraser KM, Rigano KS, Perrault JR (2019) Plasma chemistry in nesting leatherback sea turtles (*Dermochelys coriacea*) from Florida: understanding the importance of sample hemolysis effects on blood analytes. PloS One 14: e0222426. 31504062 10.1371/journal.pone.0222426 PMC6736308

[ref129] Stahl S (2006) Reptile hematology and serum chemistry. In NAVC Proceedings of the North American Veterinary Conference, p. 1673. Conference was hosted by the North American Veterinary Community (NAVC) in Orlando, FL, USA.

[ref130] Steen DA , Robinson OJ Jr (2017) Estimating freshwater turtle mortality rates and population declines following hook ingestion. Conserv Biol 31: 1333–1339. 28295579 10.1111/cobi.12926

[ref131] Steidl RJ , Hayes JP, Schauber E (1997) Statistical power analysis in wildlife research. J Wildl Manage 61: 270.

[ref132a] Stokes E , Johnson A, Rao M (2010) Monitoring Wildlife Populations for Management. Training Module 7 for the Network of Conservation Educators and Practitioners. American Museum of Natural History and the Wildlife Conservation Society. Vientiane, Lao PDR.

[ref132] Stringer EM , Harms CA, Beasley IF, Anderson ET (2010) Comparison of ionized calcium parathyroid hormone, and 25-hydroxivitamin D in rehabilitating and healthy wild green sea turtles (*Chelonia mydas*). J Herpetol Med Surg 20: 122–127.

[ref117] van de Schoot R , Depaoli S, King R, Kramer B, Märtens K, Tadesse MG, Vannucci M, Gelman A, Veen D, Willemsen J et al. (2021) Bayesian statistics and modelling. Nat Rev Methods Primers 1: 1.

[ref117a] van Straalen JP , Sanders E, Prummel MF, Sanders GT (1991) Bone-alkaline phosphatase as indicator of bone formation. Clin Chim Acta 201: 27–33. 1790624 10.1016/0009-8981(91)90021-4

[ref133] Villa C , Flint M, Bell I, Hof C, Limpus C, Gaus C (2017) Trace element reference intervals in the blood of healthy green sea turtles to evaluate exposure of coastal populations. Environ Pollut 220: 1465–1476. 27825845 10.1016/j.envpol.2016.10.085

[ref134] Wellek S , Lackner KJ, Jennen-Steinmetz C, Reinhard I, Hoffmann I, Blettner M (2014) Determination of reference limits: statistical concepts and tools for sample size calculation. Clin Chem Lab Med 52: 1685–1694. 25029084 10.1515/cclm-2014-0226

[ref135] Whiting SD , Guinea ML, Limpus CJ, Fomiatti K (2007) Blood chemistry reference values for two ecologically distinct populations of foraging green turtles, Eastern Indian Ocean. Comp Clin Path 16: 109–118.

[ref136] Wilkinson MD , Dumontier M, Aalbersberg IJ, Appleton G, Axton M, Baak A, Blomberg N, Boiten J-W, da Silva Santos LB, Bourne PE et al. (2016) The FAIR guiding principles for scientific data management and stewardship. Sci Data 3: 1–9. 10.1038/sdata.2016.18 PMC4792175 26978244

[ref137] Wood FE , Ebanks GK (1984) Blood cytology and hematology of the green sea turtle, *Chelonia-mydas*. Herpetologica 40: 331–336.

[ref138] Wood S , Scheipl F, Wood MS (2017) Package ‘gamm4’. Am Stat 45: 339.

