# Supplementary material for: Haematological and biochemical reference intervals for wild green turtles (Chelonia mydas): a Bayesian approach for small sample sizes
Source: Conserv Physiol. 2022 Jul 7;10(1):coac043. doi: 10.1093/conphys/coac043 (PMC10020984; doi:10.1093/conphys/coac043)
Supplement: suppl_coac043 [file suppl_coac043.zip › suppl_coac041.docx]

# **Supplementary Material for "Haematological and biochemical reference intervals for wild green turtles (*Chelonia mydas*): a Bayesian approach for small sample sizes"**

Sara Kophamel^1*^, Donna Rudd^^[[1]](#footnote-1)^¶^, Leigh C Ward^2¶^, Edith Shum^1¶^, Ellen Ariel^1^, Diana Mendez^3^, Jemma Starling^1^, Renee Mellers^1^, Richard K. Burchell^4^, Suzanne L Munns^1^

^1^ College of Public Health, Medical and Veterinary Sciences, James Cook University, 4811 Townsville, Queensland, Australia

^2^ School of Chemistry and Molecular Biosciences, The University of Queensland, 4072 Brisbane, Queensland, Australia

^3^ Australian Institute of Tropical Health and Medicine, James Cook University, 4811 Townsville, Queensland, Australia

^4^ North Coast Veterinary Specialist and Referral Centre, 4556 Sunshine Coast, Queensland, Australia

* Corresponding author

E-mail: [sara.kophamel@my.jcu.edu.au](mailto:sara.kophamel@my.jcu.edu.au) (SK)

## S1 Table. Methodological approaches of studies reporting (A) biochemical, (B) haematological and/or (C) blood gas parameters in green turtles (*Chelonia mydas*). Health status assessment included the presence/absence of fibropapillomatosis-like skin tumours. This table was created based on a Web of Science search using the terms “Chelonia mydas”, “green turtle”, “green sea turtle”, “blood”, “bioch*”, “haemat*”, “hemat*”, “gas”, “health”. English and non-English studies (i.e., studies in Spanish) were included (1984-2020). Studies which were not identified through these search terms might have been missed.

|  | Condition | Outcome^1^ | Sample size (n) | Location(s) | Lifestage (n)*^2^ | Health status | Internal factors | External factors | Statistical approach | Reference |
| --- | --- | --- | --- | --- | --- | --- | --- | --- | --- | --- |
|  | **(A) Biochemical parameters** | | |  |  |  |  |  |  |  |
|  | Wild | Reference values | 90* | Hawaii, U.S. | J (90) | Healthy and unhealthy | Health status, length, weight | Site | Frequentist (mean ± SD). Analysis of variance, Kruskal-Wallis and post hoc multiple pairwise comparisons reported. Reference ranges established only for wild, healthy animals, and compared to additional wild animals with fibropapillomatosis (n=56, juveniles). | Aguirre and Balazs, 2000 |
|  | Wild | Baseline values | 10* | Hawaii, U.S. | J (10) | Healthy and unhealthy | Health status |  | Frequentist (mean ± SD). Analysis of variance and general linear model reported. | Aguirre *et al*., 1995 |
|  | Wild | Blood profiles | 34 | North Carolina, U.S. | J (34) | Healthy and unhealthy | Cold stunning | Cold stunning | Frequentist (median, range). Wilcoxon rank-sum test reported. | Anderson *et al*., 2011 |
|  | Wild and captive | Reference values | 110* | Queensland, Australia | I (89)  M (21) | Healthy | Age, sex | Site, exposure to algal blooms | Frequentist (mean, range). Multivariate techniques, non-metric multidimensional scaling, and two-way nested Analysis of Similarity reported. Reference ranges established only for wild, healthy animals, and compared to additional captive animals and wild animals exposed to algal blooms (n=54, immature and mature turtles). | Arthur *et al*., 2008 |
|  | Wild and captive | Blood analytes | 55 | Georgia, U.S. | J (55) | Healthy and unhealthy | Health status, cold stunning, debilitation, length, animal (random factor) | Cold stunning, month of collection, time in rehabilitation | Frequentist (mean ± SD). Multivariate analysis of variance and linear mixed-effects models reported. | Bloodgood *et al*., 2019 |
|  | Wild | Blood profiles | 100* | Inagua Island, The Bahamas | J (100) | Healthy | Sex, length |  | Frequentist (mean ± SD, CV, range). Spearman rank correlation, analysis of variance and chi-square reported. | Bolten and Bjorndal, 1992 |
|  | Wild | Reference values | 39* | São Paulo State, Brazil | J (39) | Healthy and unhealthy | Health status, epibionts, length, weight |  | Frequentist (mean, range, 90% CI). Student t-test, Mann-Whitney U-test and correlation tests reported. Only healthy animals were included in the reference ranges, and were compared to individuals with fibropapillomatosis (n=9, juveniles). | de Mello and Alvarez, 2020 |
|  | Wild | Reference intervals | 194 | Queensland, Australia | U (194 turtles in total, predominantly immature) | Healthy and unhealthy | Health status, age class (i.e., based on length and weight), sex | Site | Frequentist (mean, range, 95% CI). Bootstrapping and multivariable regression model reported. Sample size varies according to the analysis performed. Only healthy animals were included in the reference ranges, and were compared to clinically unhealthy animals (n=25) and to animals with evidence or known history of fibropapillomatosis (n=54). | Flint *et al*., 2010 |
|  | Wild | Blood parameters | 162 | Queensland, Australia | U (predominantly juvenile sample) | Healthy | Epibionts, age class | Site | Not reported, frequentist assumed (average and range reported). | Flint *et al*., 2019 |
|  | Wild | Baseline profiles | 27* | Taiwan | SA (12)  A (15) | Healthy | Age class (i.e., length) |  | Frequentist (mean, 95% CI). Student's t-test reported. | Fong *et al*., 2010 |
|  | Wild | Reference values | 35* | Northern Territory (Australia) | SA (14)  A (21) | Healthy | Age class (i.e., length) |  | Frequentist (mean ± SD, range). "Parametric statistics", Mann-Whitney and Kruskal-Wallis tests reported. | Hamann *et al*., 2006 |
|  | Wild | Blood values | 62* | Ras Al Khaimah, United Arab Emirates | I (25)  M (36)  U (1) | Healthy | Length, sex |  | Frequentist (mean ± SD, range). Student's unpaired t-test reported. | Hasbun *et al*., 1998 |
|  | Wild | Blood parameters | 315 | Florida, U.S. | I (315) | Healthy and unhealthy | Health status, length | Site | Frequentist (least-squares mean, SE, 95% CI). Linear regression equation reported. Only total protein and haemoglobin reported. | Hirama *et al*., 2014 |
|  | Wild | Blood values | 67* | Baja California Sur, Mexico | J (49)  SA (15)  A (3) | Healthy and unhealthy | Health status, age class (i.e., length) | Site, season, year | Frequentist (mean ± SD, range). Student's t-test, analysis of variance, Mann-Whitney U-test and  Kruskal-Wallis test reported. Animals considered unhealthy included in reference ranges. | Labrada-Martagon *et al.*, 2010 |
|  | Wild | Baseline values | 28 | San Cristóbal Island, Galapagos | I (23)  A (5) | Healthy | Length, cloacal temperature | Site | Frequentist (mean ± SD, range). Student's t-test and linear regression reported. | Lewbart *et al*., 2014 |
|  | Wild | Clinical parameters | 22* | Pingtun County, Taiwan | J (22) | Unhealthy | Health status (i.e., presence or absence of fibropapillomatosis-like tumours) |  | Frequentist (mean ± SD, range). Student's t-test and Mann-Whitney  U-tests reported. Only healthy animals were included in the reference ranges, and were compared to animals with fibropapillomatosis (n=6). | Li and Chang, 2020 |
|  | Wild | Blood profiles | 127 | New South Wales, Australia | J (78.5%)  U (21.5%, presumably SA) | Unhealthy | Survival (released versus deceased animals), age class (i.e., length) | Site | Frequentist (mean ± SD). Permutational analysis of variance, chi square contingency test, Pearson correlation, and Student's t-test reported. | March *et al.*, 2018 |
|  |  |  |  |  |  |  |  |  |  |  |
|  | Wild | Blood parameters | 20* | Florida, U.S. | J (20) | Healthy and unhealthy | Health status (i.e., fibropapillomatosis-tumour score), weight, length, surface body temperature |  | Frequentist (mean, SD, median, range). Linear regression, Student's t-test and Wilcoxon test reported. 90% of the animals sampled had fibropapillomatosis. | McNally *et al*., 2020 |
|  | Wild | Blood values | 28* | Zulia, Venezuela | SA (16)  A (12) | Healthy | Age class (i.e., length) |  | Frequentist (mean ± SD, range). Student's t-test reported. | Montilla *et al.*, 2008 |
|  | Wild | Reference intervals | 99 | Culebra, Puerto Rico | J (67)  SA (32) | Healthy | Weight and length |  | Frequentist (lower and upper limits, 90% CI). Mann-Whitney U test for non-normal data reported. | Page-Karjian *et al*., 2015 |
|  | Wild | Baseline blood data | 60* | Florida, U.S. | A (60) | Healthy | Length, infection status (i.e., fibropapillomatosis) |  | Frequentist (mean ± SD, median, range, 90% CI). Multivariate framework, Cohen's kappa coefficient, correlation analysis, linear least-squares regression, and Spearman's rank order correlations reported. | Page-Karjian *et al.*, 2020 |
|  | Wild | Glucose concentra-tions | 31 | Florida, U.S. | J (31) | Healthy and unhealthy |  | Device used | Frequentist (mean ± SD, 95% CI). Passing-Bablok regression, Spearman correlation coefficient, Bland-Altman test, Welch analysis of variance and Games-Howell post hoc test reported. Only plasma glucose concentrations examined. | Perrault *et al*., 2018 |
|  | Wild | Baseline values | 59* | Isla de Aves, Venezuela | A (59) | Healthy | Length | Remigration period (as reproductive state indicator) | Frequentist (mean ± SD, range, 95% CI). Linear regression analysis, Student's t-test, Mann-Whitney U-test, Kruskal-Wallis and analysis of variance reported. | Prieto-Torres *et al*., 2013 |
|  | Wild | Baseline blood profiles | 28 | Bimini, Bahamas | J (28) | Healthy | Length | Site | Frequentist (mean ± SD). Welch's t-test, Mann-Whitney U-test, Spearman rank correlations reported. | Putillo *et al*., 2020 |
|  | Wild | Baseline data | 18* | Sistan and Baluchestan Province, Iran (Northern coast of the Sea of Oman) | A (18) | Healthy |  | Anticoagulant used | Frequentist (mean ± SD, range). Kruskal-Wallis test reported. | Sinaei *et al*., 2019 |
|  | Wild | Blood biochemistry | 14 | North Carolina, U.S. | J (14) | NA (i.e., bycaught turtles) | Cloacal body temperature, carapace area, release status (initial versus pre-release values) | Water temperature, water salinity | Frequentist (median ± SD). Analysis of covariance and Student's t-test reported. | Snoddy *et al*., 2009 |
|  | Wild | Blood values | 29 | Barbados | J (29) | Healthy |  | Food supplementation (11 animals received supplemental feeding) | Frequentist (mean ± SD). Mann-Whitney U-test and Fisher's exact test reported. | Stewart *et al*., 2016 |
|  | Wild | Blood ranges | 31* | Piura, Peru | I (31) | NA (i.e., bycaught turtles) | Length |  | Frequentist (mean ± SD, range, 95% CI). Pearson correlation test reported. | Suarez-Yana *et al*., 2016 |
|  | Wild | Reference values | 110* | Northern Territory and Ashmore Islands, Australia | I (110) | Healthy | Age class (i.e., length) | Site | Frequentist (mean ± SD, range, 95% CI). Kruskal-Wallis analysis of variance reported. Only healthy animals were included in the reference ranges and were compared to clinically unhealthy animals (n=3). | Whiting *et al*., 2007 |
|  | (CI) Confidence interval, (SD) standard deviation, (SE) standard error.  ^1^ Outcome of each study was defined based on the wording used in the corresponding Abstracts  * Indicates low sample size for establishing reference ranges (n<120)  *^2^ Lifestage: (J) Juvenile, (SA) Subadult, (A) Adult, (I) Immature, (M) Mature, (U) Unknown. | | | | | | | | | |

|  | Condition | Outcome^1^ | Sample size (n) | Location(s) | Lifestage (n)*^2^ | Health status | Internal factors | External factors | Statistical approach | Reference |
| --- | --- | --- | --- | --- | --- | --- | --- | --- | --- | --- |
|  | **(B) Haematological parameters** (please note that some of these studies only reported packed cell volume, PCV, and focused on biochemical parameters instead) | | | | | | | | |  |
|  | Wild | Baseline values | 10* | Hawaii, U.S. | J (10) | Healthy and unhealthy | Health status |  | Frequentist (mean ± SD). Analysis of variance and general linear model reported. | Aguirre *et al*., 1995 |
|  | Wild | Blood profiles | 34 | North Carolina, U.S. | J (34) | Healthy and unhealthy | Cold stunning | Cold stunning | Frequentist (median, range). Wilcoxon rank-sum test reported. | Anderson *et al*., 2011 |
|  | Wild and captive | Blood analytes | 55 | Georgia, U.S. | J (55) | Healthy and unhealthy | Health status, cold stunning, debilitation, length, animal (random factor) | Cold stunning, month of collection, time in rehabilitation | Frequentist (mean ± SD). Multivariance analysis of variance and linear mixed-effects models reported. | Bloodgood *et al.*, 2019 |
|  | Wild | Blood profiles | 106* | Inagua Island, The Bahamas | J (106) | Healthy | Sex, length |  | Frequentist (mean ± SD, CV, range). Spearman rank correlation, analysis of variance and chi-square reported. Only PCV reported. | Bolten and Bjorndal, 1992 |
|  | Wild | Reference values | 39* | São Paulo State, Brazil | J (39) | Healthy and unhealthy | Health status, epibionts, length, weight |  | Frequentist (mean, range, 90% CI). Student t-test, Mann-Whitney U-test and correlation tests reported. Only healthy animals were included in the reference ranges, and were compared to individuals with fibropapillomatosis (n=9, juveniles). | de Mello and Alvarez, 2020 |
|  | Wild | Reference intervals | 178 | Queensland, Australia | I (105)  M (32)  U (41) | Healthy and unhealthy | Health status, age class (i.e., based on length and weight), sex | Site | Frequentist (mean, range, 95% CI). Bootstrapping and multivariable regression model reported. Sample size varies according to the analysis performed. Only healthy animals were included in the reference ranges, and were compared to clinically unhealthy animals (n=25) and to animals with evidence or known history of fibropapillomatosis (n=54). | Flint *et al*., 2010 |
|  | Wild | Blood parameters | 162 | Queensland, Australia | U (predominantly juvenile sample) | Healthy | Epibionts, age class, | Site | Not reported, frequentist assumed (average and range reported). | Flint *et al*., 2019 |
|  | Wild | Reference values | 30* | Zulia, Venezuela | J (2)  SA (16)  A (12) | Healthy and unhealthy | NA | NA | Frequentist (median ± SD, range). | Fuenmayor *et al*., 2006 |
|  | Wild | Baseline profiles | 27* | Taiwan | SA (12)  A (15) | Healthy | Age class (i.e., length) |  | Frequentist (mean, 95% CI). Student's t-test reported. Only PCV reported. | Fong *et al*., 2010 |
|  | Wild | Blood parameters | 328 | Florida, U.S. | I (328) | Healthy and unhealthy | Health status, length | Site | Frequentist (least-squares mean, SE, 95% CI). Linear regression equation reported. Only PCV reported. | Hirama *et al*., 2014 |
|  | Wild | Baseline values | 84* | Yucatan, Mexico |  | Healthy and unhealthy | Health status |  | Not reported, frequentist assumed (range reported). Only healthy animals were included in the reference ranges, and were compared to clinically unhealthy animals (n=16). | Lara-Ucetal *et al*., 2016 |
|  | Wild | Baseline values | 28 | San Cristóbal Island, Galapagos | I (23)  A (5) | Healthy | Length, cloacal temperature | Site | Frequentist (mean ± SD, range). Student's t-test and linear regression reported. | Lewbart *et al*., 2014 |
|  | Wild | Clinical parameters | 22* | Pingtun County, Taiwan | J (22) | Unhealthy | Health status (i.e., presence or absence of fibropapillomatosis-like tumours) |  | Frequentist (mean ± SD, range). Student's t-test and Mann-Whitney U-tests reported. Only healthy animals were included in the reference ranges, and were compared to animals with fibropapillomatosis (n=6). | Li and Chang, 2020 |
|  | Wild | Blood profiles | 127 | New South Wales, Australia | J (78.5%)  U (21.5%, presumably SA) | Unhealthy | Survival (released versus deceased animals), age class (i.e., length) | Site | Frequentist (mean ± SD). Permutational analysis of variance, chi square contingency test, Pearson correlation, and Student's t-test reported. | March *et al*., 2018 |
|  | Wild | Blood parameters | 20* | Florida, U.S. | J (20) | Healthy and unhealthy | Health status (i.e., fibropapillomatosis-tumour score), weight, length, surface body temperature |  | Frequentist (mean, SD, median, range). Linear regression, Student's t-test and Wilcoxon test reported. 90% of the animals sampled had fibropapillomatosis. Only PCV reported. | McNally *et al.*, 2020 |
|  | Wild | Reference intervals | 99 | Culebra, Puerto Rico | J (67)  SA (32) | Healthy | Weight and length |  | Frequentist (lower and upper limits, 90% CI). Mann-Whitney U test for non-normal data reported. | Page-Karjian, *et al.,* 2015 |
|  | Wild | Baseline blood data | 60* | Florida, U.S. | A (60) | Healthy | Length, infection status (i.e., fibropapillomatosis) |  | Frequentist (mean ± SD, median, range, 90% CI). Multivariate framework, Cohen's kappa coefficient, correlation analysis, linear least-squares regression, and Spearman's rank order correlations reported. | Page-Karjian *et al*., 2020 |
|  | Wild | Blood values | 64* | Isla de Aves, Venezuela | A (64) | Healthy and unhealthy | Length |  | Frequentist (mean ± SD, range, 95% CI). Pearson and Spearman correlations reported. | Prieto-Torres *et al*., 2012 |
|  | Wild | Blood values | 29* | Baja California Sur, Mexico | SA (29) | Healthy |  |  | NA. Mean ± SD reported. | Reséndiz *et al*., 2018 |
|  | Wild | Blood profile | 45 | São Paulo, Brazil | J (45) | Unhealthy | Health status (i.e., fibropapillomatosis-tumour score) |  | Frequentist (mean ± SD). Kruskal-Wallis test reported. | Rossi *et al*., 2009 |
|  | Wild | Reference values | 72* | Ras Al-Khaima, United Arab Emirates | J (13)  SA (14)  A (45) | Healthy | Age class (i.e., length), sex |  | Frequentist (mean ± SEM, range). Student's t-test reported. | Samour *et al*., 1998 |
|  | Wild | Baseline data | 18* | Sistan and Baluchestan Province, Iran (Northern coast of the Sea of Oman) | A (18) | Healthy |  | Anticoagulant used | Frequentist (mean ± SD, range). Kruskal-Wallis test reported. | Sinaei *et al*., 2019 |
|  | Wild | Blood values | 29 | Barbados | J (29) | Healthy |  | Food supplementation (11 animals received supplemental feeding) | Frequentist (mean ± SD). Mann-Whitney U-test and Fisher's exact test reported. | Stewart *et al*., 2016 |
|  | Wild | Blood ranges | 31* | Piura, Peru | I (31) | NA (i.e., bycaught turtles) | Length |  | Frequentist (mean ± SD, range, 95% CI). Pearson correlation test reported. | Suarez-Yana *et al*., 2016 |
|  | Wild | Reference values | 110* | Northern Territory and Ashmore Islands, Australia | I (110) | Healthy | Age class (i.e., length) | Site | Frequentist (mean ± SD, range, 95% CI). Kruskal-Wallis analysis of variance reported. Only healthy animals were included in the reference ranges, and were compared to clinically unhealthy animals (n=3). | Whiting *et al*., 2007 |
|  | Captive | Haematolo-gical parameters | 51* | Grand Cayman, Cayman Islands | J (31)  A (20) | Healthy | Age (i.e., months, years) |  | Frequentist (mean ± SD, range). Analysis of variance reported. | Wood and Ebanks, 1984 |
|  | Wild | Morphologic characteris-tics | 26* | Hawaii, U.S. | J (26) | Healthy |  |  | Frequentist (mean ± SD, range). Student's t-test and Mann-Whitney U-test reported. | Work *et al*., 1998 |
|  | Wild | Blood profiles | 47 | São Paulo, Brazil | J (47) | Unhealthy (i.e., turtles in rehabilitation) | Presence/absence of fibropapillomatosis-  like tumours |  | Frequentist (mean ± SD, range). Student's t-test and Mann-Whitney U-test reported. | Zwarg *et al*., 2014 |

(CI) Confidence interval, (SD) standard deviation, (SE) standard error. ^1^ Outcome of each study was defined based on the wording used in the corresponding Abstracts

* Indicates low sample size (n<120). *^2^ Lifestage: (J) Juvenile, (SA) Subadult, (A) Adult, (I) Immature, (M) Mature, (U) Unknown.

|  | Condition | Outcome^1^ | Sample size (n) | Location(s) | Lifestage (n)*^2^ | Health status | Internal factors | External factors | Statistical approach | Reference |
| --- | --- | --- | --- | --- | --- | --- | --- | --- | --- | --- |
|  | **(C) Blood gases** | | |  |  |  |  |  |  |  |
|  | Wild | Blood profiles | 34 | North Carolina, U.S. | J (34) | Healthy and unhealthy | Cold stunning | Cold stunning | Frequentist (median, range). Wilcoxon rank-sum test reported. | Anderson *et al*., 2011 |
|  | Wild | Baseline values | 28 | San Cristóbal Island, Galapagos | I (23)  A (5) | Healthy | Length, cloacal temperature | Site | Frequentist (mean ± SD, range). Student's t-test and linear regression reported. | Lewbart *et al.*, 2014 |
|  | Wild | Clinical parameters | 22* | Pingtun County, Taiwan | J (22) | Unhealthy | Health status (i.e., presence or absence of fibropapillomatosis-like tumours) |  | Frequentist (mean ± SD, range). Student's t-test and Mann-Whitney U-tests reported. Only healthy animals were included in the reference ranges and were compared to animals with fibropapillomatosis (n=6). | Li and Chang, 2020 |
|  | Wild | Blood parameters | 20* | Florida, U.S. | J (20) | Healthy and unhealthy | Health status (i.e., fibropapillomatosis-tumour score), weight, length, surface body temperature |  | Frequentist (mean, SD, median, range). Linear regression, Student's t-test and Wilcoxon test reported. 90% of the animals sampled had fibropapillomatosis. | McNally *et al*., 2020 |
|  | Wild | Reference intervals | 99 | Culebra, Puerto Rico | J (67)  SA (32) | Healthy | Weight and length |  | Frequentist (lower and upper limits, 90% CI). Mann-Whitney U test for non-normal data reported. | Page-Karjian *et al.*, 2015 |
|  | (CI) Confidence interval, (SD) standard deviation, (SE) standard error.  ^1^ Outcome of each study was defined based on the wording used in the corresponding Abstracts  * Indicates low sample size (n<120)  *^2^ Lifestage: (J) Juvenile, (SA) Subadult, (A) Adult, (I) Immature, (M) Mature, (U) Unknown. | | | | | | | | | |
|  |  |  |  |  |  |  |  |  |  |  |
|  |  |  |  |  |  |  |  |  |  |  |
|  |  |  |  |  |  |  |  |  |  |  |

**S2 Table. Haematological (A) and biochemical (B) measured analyte values of green sea turtles (*Chelonia mydas*, *n*=97).** Turtles were captured at foraging grounds in Howick Group of Islands (Howick) and Townsville region (Townsville), located in North Queensland, Australia.

**(A) Haematological analyte values**

| Analyte (unit) | Location (*n*) | Mean ± 2SD | Lowest value | Highest value |
| --- | --- | --- | --- | --- |
| Packed cell volume (%) | Townsville (24) | 25.9 ± 16.4 | 14.5 | 41.4 |
| Heterophils (%) | Townsville (28) | 48.8 ± 22.2 | 30 | 78 |
| Lymphocytes (%) | Townsville (28) | 42.3 ± 20.6 | 19 | 65 |
| Monocytes (%) | Townsville (26) | 3.7 ± 3.9 | 1 | 8 |
| Eosinophils (%) | Townsville (27) | 4.1 ± 6.1 | 0 | 10.5 |
| Basophils (%) | Townsville (28) | 0.1 ± 0.7 | 0 | 1 |
| Heterophil:  Lymphocyte ratio (ratio) | Townsville (28) | 1.3 ± 1.5 | 0.5 | 4 |

(*n*) Sample size; (SD) Standard deviation

**(B) Biochemical analyte values**

| Analyte (unit) | Captivity status (*n*) | Mean ± 2SD | Lowest value | Highest value |
| --- | --- | --- | --- | --- |
| Albumin (g/L) | Townsville (40) | 9.2 ± 7.0 | 2 | 17 |
|  | Howick (57) | 13.5 ± 5.8 | 5 | 21 |
| Alkaline phosphatase (U/L) | Townsville (40) | 14.1 ± 14.0 | 1.5 | 34.1 |
|  | Howick (57) | 20.5 ± 23.8 | 4.7 | 66.6 |
| Aspartate transaminase (U/L) | Townsville (37) | 212.9 ± 127.0 | 58.5 | 347.3 |
|  | Howick (56) | 180.2 ± 124.3 | 64.5 | 417.1 |
| Total bilirubin (µmol/L) | Townsville (37) | 1.8 ± 1.2 | 0.7 | 3.2 |
|  | Howick (56) | 2.2 ± 1.4 | 0.7 | 4.6 |
| Calcium (mmol/L) | Townsville (40) | 1.7 ± 0.7 | 1 | 2.6 |
|  | Howick (57) | 2.3 ± 1.4 | 0.9 | 4.7 |
| Chloride (mmol/L) | Townsville (37) | 113.9 ± 14.1 | 96.5 | 126.5 |
|  | Howick (57) | 111.4 ± 9.7 | 100.8 | 123.5 |
| Cholesterol (mmol/L) | Townsville (39) | 2.1 ± 2.3 | 0.3 | 4.4 |
|  | Howick (55) | 4.3 ± 3.0 | 1.1 | 7.2 |
| Creatine kinase (U/L) | Townsville (38) | 1136.6 ± 1138.4 | 188.4 | 2595 |
|  | Howick (56) | 1369.5 ± 1724.2 | 277.2 | 3971.8 |
| Creatinine (µmol/L) | Townsville (21) | 4.4 ± 5.2 | 1 | 10 |
|  | Howick (47) | 4.7 ± 5.0 | 1 | 11 |
| Globulins (g/L) | Townsville (40) | 22.5 ± 13.2 | 8 | 38.2 |
|  | Howick (57) | 32.1 ± 14.0 | 13.2 | 49.1 |
| Glucose (mmol/L) | Townsville (40) | 5.6 ± 3.0 | 2.9 | 11.2 |
|  | Howick (57) | 5.2 ± 2.1 | 2.8 | 8.3 |
| Lactate dehydrogenase (U/L) | Townsville (38) | 163.2 ± 126.9 | 55.9 | 362.1 |
|  | Howick (57) | 192.9 ± 155.5 | 95.3 | 467.4 |
| Magnesium (mmol/L) | Townsville (40) | 3.8 ± 1.4 | 1.9 | 4.9 |
|  | Howick (57) | 4.3 ± 1.1 | 3.2 | 5.6 |
| Phosphate (mmol/L) | Townsville (40) | 2.3 ± 1.1 | 1.3 | 3.7 |
|  | Howick (57) | 1.8 ± 1.2 | 0.6 | 4.2 |
| Total protein (g/L) | Townsville (40) | 31.7 ± 18.3 | 10.0 | 55.2 |
|  | Howick (57) | 45.6 ± 18.4 | 19.8 | 69.1 |
| Potassium (mmol/L) | Townsville (40) | 4.0 ± 1.6 | 1.8 | 5.9 |
|  | Howick (57) | 4.7 ± 1.5 | 3.4 | 6.6 |
| Sodium (mmol/L) | Townsville (33) | 153.3 ± 8.5 | 141.2 | 161.8 |
|  | Howick (57) | 154.4 ± 10.0 | 140.6 | 167.3 |
| Triglycerides (mmol/L) | Townsville (39) | 0.5 ± 0.7 | 0.2 | 1.8 |
|  | Howick (55) | 1.3 ± 1.6 | 0.1 | 3.2 |
| Urea (mmol/L) | Townsville (36) | 5.1 ± 11.9 | 0.3 | 25.8 |
|  | Howick (57) | 3.4 ± 10.3 | 0.1 | 28.8 |
| Uric acid (mmol/L) | Townsville (39) | 0.1 ± 0.0 | 0.01 | 0.11 |
|  | Howick (55) | 0.1 ± 0.1 | 0.01 | 0.21 |
| Ca:P ratio (ratio) | Townsville (40) | 0.8 ± 0.5 | 0.4 | 1.4 |
|  | Howick (57) | 1.5 ± 1.7 | 0.4 | 4.3 |
| Albumin:Globulin ratio (ratio) | Townsville (40) | 0.4 ± 0.3 | 0.2 | 0.8 |
|  | Howick (57) | 0.4 ± 0.2 | 0.3 | 0.8 |

(*n*) Sample size; (SD) Standard deviation


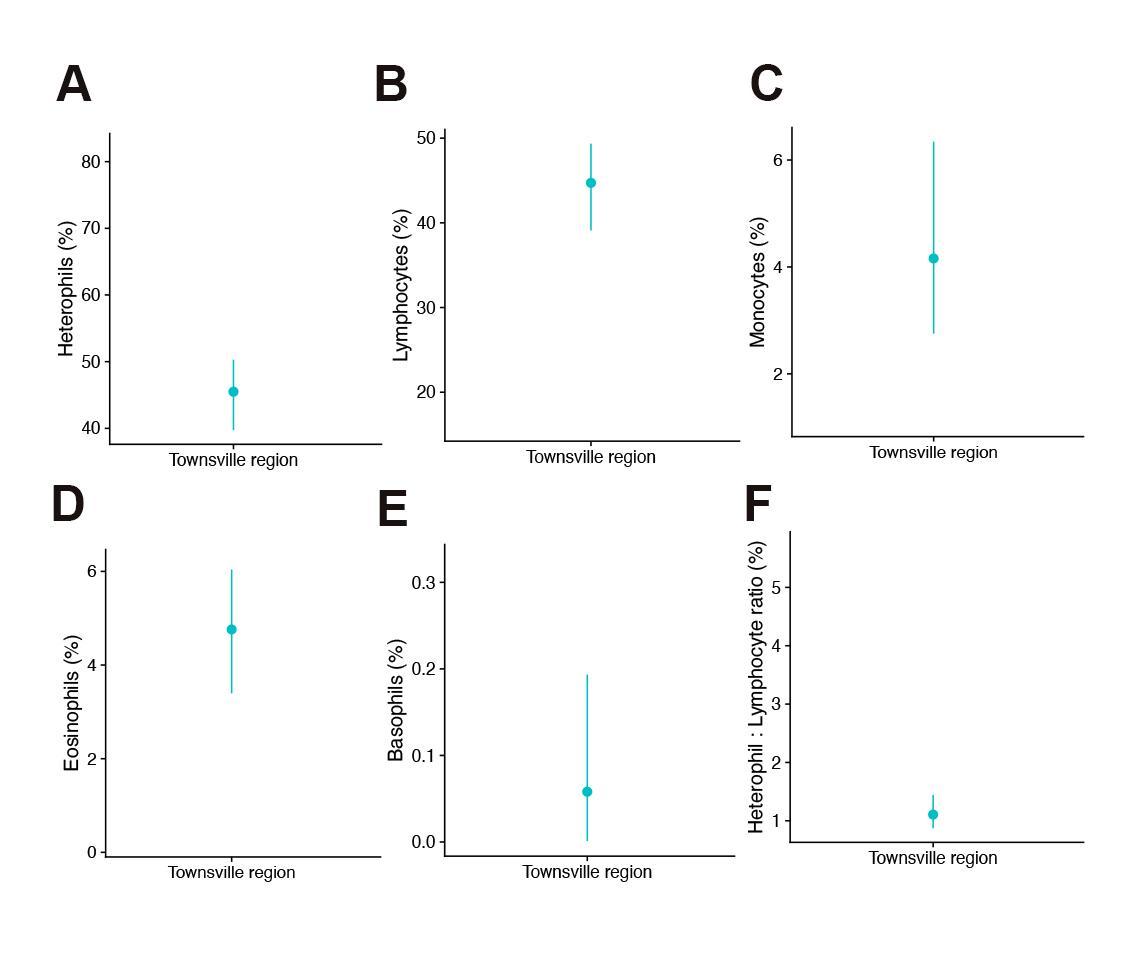
**S1 Figure. Predicted haematological reference intervals for green turtles (*Chelonia mydas*) in Townsville region (*n*=28).** Haematological parameters measured: (A) heterophils, (B) lymphocytes, (C) monocytes, (D) eosinophils, (E) basophils, and (F) heterophil:lymphocyte ratio. Symbols represent the estimated marginal mean (EMM), and error bars indicate the % higher posterior density credible intervals (HPDCI) lower and upper limits (analogous to mean +/- 2 SD).

**S2 Figure. Predicted biochemical reference intervals for green turtles (*Chelonia mydas*) in North Queensland, Australia (*n*=97).** Biochemical parameters measured: (A) albumin (g/L), (B) alkaline phosphatase (U/L), (C) aspartate transaminase (U/L), (D) total bilirubin (µmol/L), (E) calcium (mmol/L), (F) chloride (mmol/L), (G) cholesterol (mmol/L), (H) creatine kinase (U/L), (I) creatinine (µmol/L), (J) glucose (mmol/L), (K) lactate dehydrogenase (U/L), (L) magnesium (mmol/L), (M) phosphate (mmol/L), (N) potassium (mmol/L), (O) total protein (g/L), (P) sodium (mmol/L), (Q) triglycerides (mmol/L), (R) urea (mmol/L), (S) uric acid (mmol/L), and (T) Globulin:Albumin ratio. Symbols represent the estimated marginal mean (EMM), and error bars indicate the % higher posterior density credible intervals (HPDCI) lower and upper limits (analogous to mean +/- 2 SD).

**S3 Table.** **Trends, effect sizes and % differences in wild green turtle (*Chelonia mydas*) biochemical and haematological reference intervals (n=97) between our study and blood values, parameters, or intervals reported by other studies.**

| Parameter | Comparative study | Trend in our study | Effect size  (Hedge's g) | Mean % difference between our study and comparison study |
| --- | --- | --- | --- | --- |
| **Haematological analytes** | |  |  |  |
| Heterophils | Lewbart *et al*., 2014 | Higher ↑ | 1.7 | 177 % |
|  | Samour *et al*., 1998 | Lower ↓ | 0.8 | 11 % |
| Lymphocytes | Samour *et al*., 1998 | Higher ↑ | 1.7 | 273 % |
|  | March *et al*., 2018 | Higher ↑ | 2.2 | 180 % |
| Eosinophils | Lewbart *et al*., 2014 | Lower ↓ | 3.5 | 77 % |
|  | Samour *et al*., 1998 | Lower ↓ | 1.9 | 48 % |
| Monocytes | Lewbart *et al*., 2014 | Lower ↓ | 2.0 | 66 % |
|  | March *et al*., 2018 | Lower ↓ | 1.4 | 49 % |
|  | |  |  |  |
| **Biochemical analytes** | |  |  |  |
|  |  |  |  |  |
| Creatinine | Flint *et al*., 2010 | Lower ↓ | NA* | NA* |
|  | Hamann *et al*., 2006 | Lower ↓ | 4.8 | 82 % |
|  | Whiting *et al*., 2007 | Lower ↓ | 6.1 | 85 % |
|  | Aguirre and Balazs, 2000 | Lower ↓ | 2.2 | 75 % |
|  | Bolten and Bjorndal, 1992 | Lower ↓ | 5.7 | 90 % |
| Urea | Hamann *et al*., 2006 | Higher ↑ | 1.7 | 260 % |
| Uric acid | Whiting *et al*., 2007 | Lower ↓ | 1.5 | 44 % |
| Serum creatinine kinase | March *et al*., 2018 | Lower ↓ | 3.8 | 73 % |
| Serum glucose | Hamann *et al*., 2006 | Higher ↑ | 1.6 | 229 % |
| Phosphate | Flint *et al*., 2010 | Lower ↓ | NA* | NA* |
| Magnesium | Whiting *et al*., 2007 | Higher ↑ | 0.9 | 61 % |
| Sodium | Bolten and Bjorndal, 1992 | Lower ↓ | 0.4 | 11 % |

* Comparison of studies was not possible due to missing statistical information. Hedge's g was used for effect size calculation.

**S1 Appendix. Additional results**

Correlations between variables were assessed using Pearson's and Spearman's correlation coefficient analyses (strong correlation assumed when p<0.05 and r>0.5). Strong positive correlations (p<0.05 and r>0.5) in haematological analytes were found between: (1) PCV and heterophil to lymphocyte ratio, albumin, alkaline phosphatase, and cholesterol; (2) heterophils and phosphate, and triglycerides; and (3) lymphocytes and magnesium. Strong negative correlations were found between: (1) heterophils and lymphocytes, eosinophils, and magnesium; (2) lymphocytes and heterophils; and (3) eosinophils and heterophils. Strong correlations (p<0.05 and r>0.5) were also found between other biochemical analytes. Positive correlation was found between (1) albumin and PCV, calcium, cholesterol, total protein, triglycerides, and potassium; (2) alkaline phosphatase and PCV, cholesterol and triglycerides; (3) total bilirubin and total protein; (4) calcium and albumin, magnesium, and total protein; (5) cholesterol and PCV, albumin, alkaline phosphatase, total protein, triglycerides, and potassium; (6) phosphate and heterophils; (7) lactate dehydrogenase and potassium; (8) magnesium and lymphocytes, calcium, total protein and potassium; (9) total protein and albumin, total bilirubin, calcium, cholesterol, magnesium, triglycerides, and potassium; (10) triglycerides and heterophils, albumin, alkaline phosphatase, cholesterol, and total protein; (11) uric acid and potassium; (12) sodium and chloride; (13) potassium and albumin, cholesterol, lactate dehydrogenase, magnesium, total protein, and uric acid; and (14) chloride and sodium. Negative correlations were found between: (1) phosphate and magnesium; (2) magnesium and heterophils, and phosphate; (3) total protein and urea; and (4) urea and calcium, and total protein. Location was not strongly correlated with other influencing parameters included in the model.

## S2 Appendix. Description of the parameters examined and of the codes used in the dataset. The dataset used for the analyses is available as a spreadsheet saved in MS Excel (.xlsx), Open Document (.ods), and Comma-separated values (.csv) formats in Research Data Australia, at [https://doi.org/10.25903/9rm7-k267](https://aus01.safelinks.protection.outlook.com/?url=https%3A%2F%2Fdoi.org%2F10.25903%2F9rm7-k267&data=05%7C01%7Csara.kophamel%40my.jcu.edu.au%7Ceede1e95d1a948edf97808da2270d11b%7C2eba4cf8af764db3bcaf81b5592535ef%7C0%7C0%7C637860165213286113%7CUnknown%7CTWFpbGZsb3d8eyJWIjoiMC4wLjAwMDAiLCJQIjoiV2luMzIiLCJBTiI6Ik1haWwiLCJXVCI6Mn0%3D%7C3000%7C%7C%7C&sdata=A%2Bo2iTbQbVM9xqnhZS5VaFTQh29%2FPu1K0rZyxXqJM%2FA%3D&reserved=0) [DOI: 10.25903/9rm7-k267] (Kophamel and Munns, 2022).

- id: Animal identification number
- date: Date the sample was collected (day/month/year).
- season: Season in which the sample was collected (dry/wet)
- location: Location in which the animals were captured or sourced from. "combe_reef" and "ingram_reef" are locations in Howick Group of Islands; "cockle_bay" and "toolakea" are locations in the Townsville region
- location_merged: Summarised location in which the animals were captured or sourced from. "howicks" refers to Howick Group of Islands; "tsv" refers to Townsville region
- location_merged_num: Numeric code of the variable "location_merged". (2) tsv; (3) howicks
- recap: Record of inter-season (isr) or within-season (wsr) recapture. (p) denotes an animal which was captured for the first time
- lifestage: Life stage of the animals sampled. (j) juvenile; (sa) subadult; (a) adult
- sex: Sex of the animals sampled. (f) female; (m) male; (i) undetermined
- sex_num: Numeric code of the variable "sex". (1) female; (2) male; (3) undetermined
- ccl: Curved carapace length (cm)
- scl: Straight carapace length (cm)
- weight: Total body weight (kg)
- bci: Body condition index
- ph_ex: Evidence of physical examination. (1) yes
- temp_air: Air temperature (ºC)
- temp_cloaca: Cloacal temperature (ºC)
- postfeeding_h: Postprandial time (hours)
- blood: Evidence of blood analysis. (1) yes
- istat: Additional blood analysed using an i-STAT blood gas analyser (unpublished data, which is part of a separate research study)
- bia_postcapt_h: Hours after capture
- smear: Evidence of blood smear. (1) yes; (0) no
- biochem: Evidence of biochemical analysis. (1) yes
- pcv: Packed cell volume
- wbc_het: Heterophils (%)
- wbc_lymph: Lymphocytes (%)
- wbc_mono: Monocytes (%)
- wbc_eosin: Eosinophils (%)
- wbc_baso: Basophils (%)
- wbc_azu: Presence of azurophil-like cells, not included in the analysis. (T) true; (F) false.
- wbc_ratio_het_lymph: Heterophil : Lymphocyte ratio
- bc_Alb: Albumin (g/L)
- bc_ALP: Alkaline phosphatase (U/L)
- bc_AST: Aspartate transaminase (U/L)
- bc_TBILC: Total bilirubin (µmol/L)
- bc_CALA: Calcium (mmol/L)
- bc_CO2: Carbon dioxide (mmol/L). Not included in the analysis
- bc_Cl: Chloride (mmol/L)
- bc_CHOL: Cholesterol (mmol/L)
- bc_CK: Creatine kinase (U/L)
- bc_CRE: Creatinine (µmol/L)
- bc_glob: Globulins (g/L)
- bc_GLUC: Glucose (mmol/L)
- bc_LDH: Lactate dehydrogenase (U/L)
- bc_MG: Magnesium (mmol/L)
- bc_PHOS: Phosphate (mmol/L)
- bc_K: Potassium (mmol/L)
- bc_TP: Total protein (g/L)
- bc_Na: Sodium (mmol/L)
- bc_TRIG: Triglycerides (mmol/L)
- bc_UREA: Urea (mmol/L)
- bc_URIC: Uric acid (mmol/L)
- bc_CaP: Calcium : Phosphate ratio
- bc_AlbGlob: Albumin : Globulin ratio

##

##
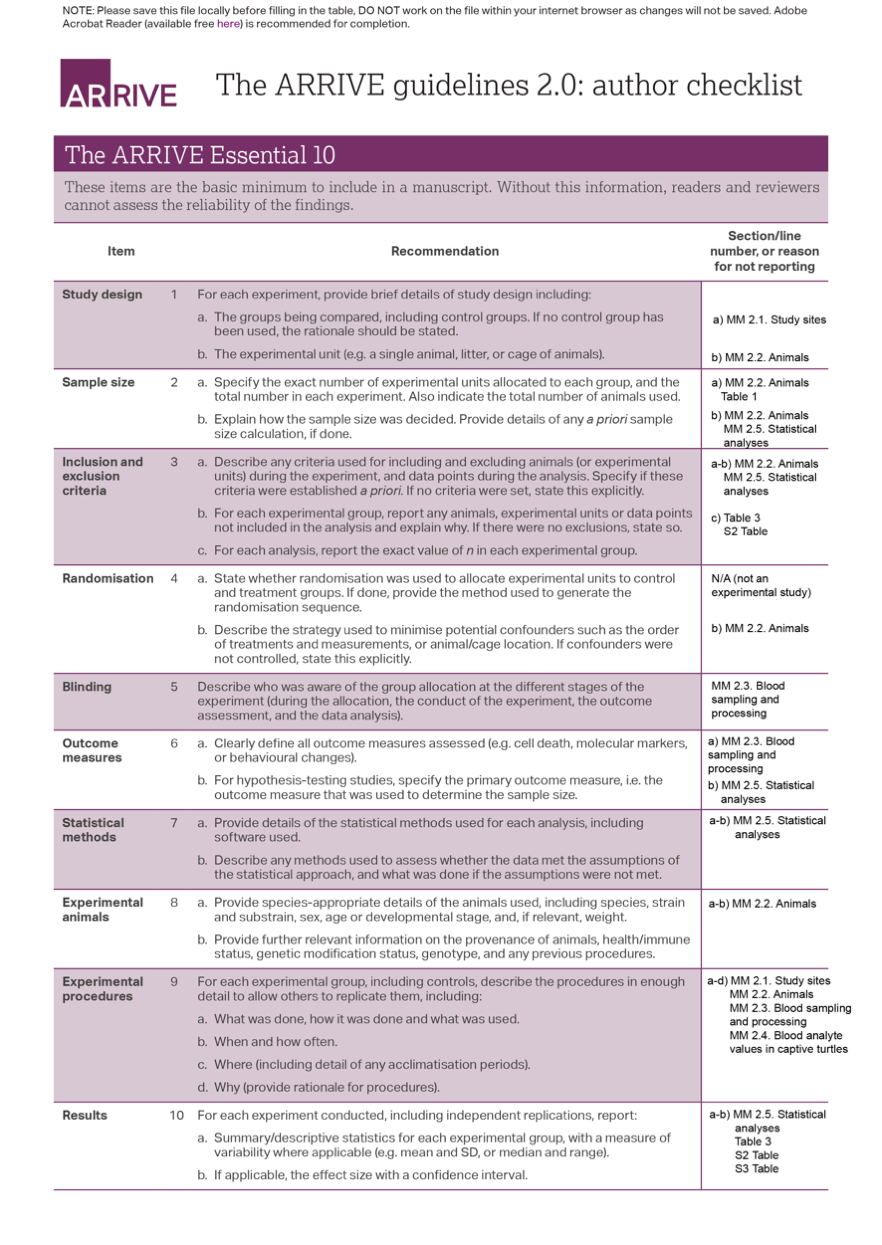

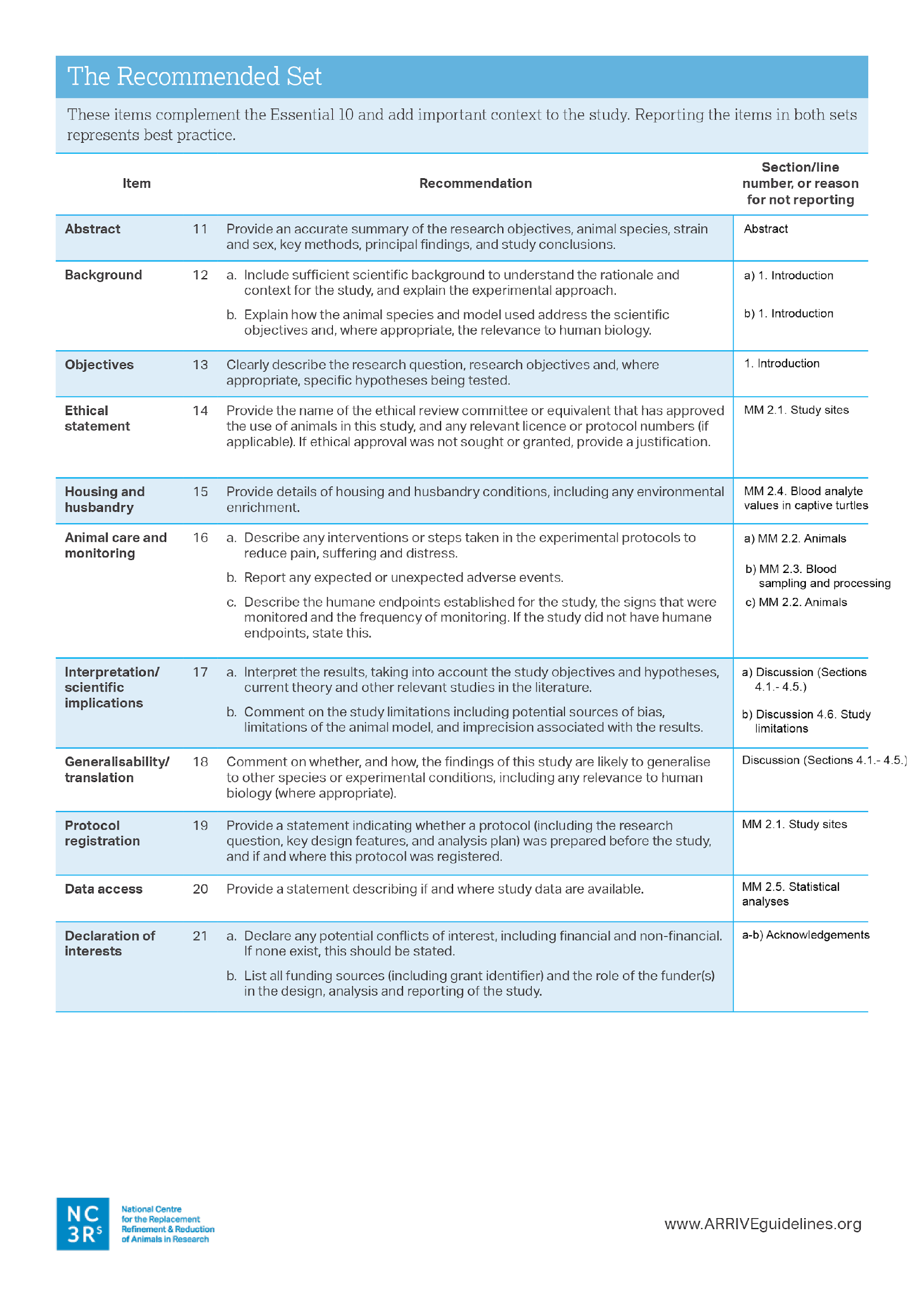
S3 Appendix. ARRIVE guidelines (version 2.0) to improve the reporting of animal research in our study. Essential and recommended set. Sourced from Percie du Sert *et al*. (2020).

## S4 Appendix. Analytical principles of the analytes measured. All reagents were purchased for use on a Beckman AU480 automated biochemistry analyser (purchased from Beckman Coulter Australia). Calibration: Beckman Coulter System Cal and Quality Control Beckman Coulter Control 1 and Control 2.

| Analyte | Analytical Principle | Catalogue Number |
| --- | --- | --- |
| Albumin | This Albumin method is a modification of the Doumas ^2^ and Rodkey ^1^ procedures utilizing a different buffering system. At pH 4.2, bromocresol green reacts with albumin to form an intense green complex. The absorbance of the albumin-BCG complex is measured bichromatically (600/800nm) and is proportional to the albumin concentration in the sample. | OSR6022 |
| Alkaline Phosphatase (ALP) | This ALP procedure is based on the method developed by Bowers and McComb^3^ and has been formulated as recommended by the AACC and IFCC. Alkaline phosphatase activity is determined by measuring the rate of conversion of p-nitro-phenylphosphate (pNPP) in the presence of 2-amino-2-methyl-1-propanol (AMP) at pH 10.4. | OSR6504 |
| Aspartate Amino Transferase (AST) | This AST procedure is a modification of the methodology recommended by the IFCC.^4^ In this method, aspartate aminotransferase (AST) catalyzes the transamination of aspartate and α-oxoglutarate, forming L-glutamate and oxalacetate. The oxalacetate is then reduced to L-malate by malate dehydrogenase, while NADH is simultaneously converted to NAD+. The decrease in absorbance due to the consumption of NADH is measured at 340 nm and is proportional to the AST activity in the sample. | OSR6509 |
| Total Bilirubin | This Total Bilirubin Reagent is a variation of the classical diazo method described by Ehrlich in 1883 ^5^. A stabilized diazonium salt, 3,5 dichlorophenyldiazonium tetrafluoroborate (DPD), reacts with bilirubin to form azobilirubin which absorbs at 570/660 nm. Caffeine and a surfactant are used as reaction accelerators.  The absorbance at 570/660 nm is proportional to the bilirubin concentration in the sample. A separate serum blank is performed to eliminate endogenous serum2 interferences. | OSR6512 |
| Calcium | This Calcium procedure is based on calcium ions (Ca2+) reacting with Arsenazo III (2,2’-[1,8-Dihydroxy-3,6-disulphonaphthylene-2,7-bisazo]-bisbenzenear-sonic acid) to form an intense purple colored complex.^6,7^ The absorbance of the Ca-Arsenazo III complex is measured bichromatically at 660/700 nm. | OSR65117 |
| Chloride | Chloride is measured using an ion selective electrode. |  |
| Cholesterol | In this procedure cholesterol esters in a sample are hydrolysed by cholesterol esterase (CHE). The free cholesterol produced is oxidised by cholesterol oxidase (CHO) to cholestene-3-one with the simultaneous production of hydrogen peroxide (H2O2), which oxidatively couples with 4-aminoantipyrine and phenol in the presence of peroxidase  (POD) to yield a chromophore. The red quinoneimine dye formed can be measured spectrophotometrically at 540/600 nm as an increase in absorbance.^8^ | OSR6216 |
| Creatine Kinase | Utilizing a modification of the IFCC method.^9,10^ CK reversibly catalyzes the transfer of a phosphate group from creatine phosphate to adenosine diphosphate (ADP) to give creatine and adenosine triphosphate (ATP) as products. The ATP formed is used to produce glucose-6-phosphate and ADP from glucose. This reaction is catalyzed by hexokinase (HK) which requires magnesium ions for maximum activity. The glucose-6-phosphate is oxidized by the action of the enzyme glucose-6-phosphate dehydrogenase (G6P-DH) with simultaneous reduction of the coenzyme nicotinamide adenine dinucleotide (NADP) to give NADPH and 6-phosphogluconate. The rate of increase of absorbance at 340/660 nm due to the formation of NADPH is directly proportional to the activity of CK in the sample. | OSR6279 |
| Creatinine | This Creatinine procedure is a kinetic modification of the Jaffe procedure,^11^ in which creatinine reacts with picric acid at alkaline pH to form a yellow/orange complex. The rate of change in absorbance at 520/800nm is proportional to the creatinine concentration in the sample. | OSR6678 |
| Glucose | In this procedure, glucose is phosphorylated by hexokinase (HK) in the presence of adenosine triphosphate (ATP) and magnesium ions to produce glucose-6-phosphate (G-6-P) and adenosine diphosphate (ADP). Glucose-6-phosphate dehydrogenase (G6P-DH) specifically oxidizes G-6-P to 6-phosphogluconate with the concurrent reduction of nicotinamide adenine dinucleotide (NAD+) to nicotinamide adenine dinucleotide, reduced (NADH). The change in absorbance at 340/380 nm is proportional to the amount of glucose present in the sample. | OSR6621 |
| Lactate Dehydrogenase (LDH) | The LDH procedure employs a modification of the method of Wacker *et al.*^13,14^ which utilizes the forward reaction of lactate to pyruvate and NADH catalyzed by LD. NADH strongly absorbs light at 340 nm, whereas NAD does not. The rate of change of absorbance at 340 nm is directly proportional to the LD activity in the sample. | OSR6227 |
| Magnesium | This Magnesium procedure utilizes a direct method in which magnesium forms a colored complex with xylidyl blue in a strongly basic solution. The color produced is measured bichromatically at 520/800 nm and is proportional to the magnesium concentration.^15,16^ | OSR6189 |
| Inorganic Phosphate | This method for the determination of phosphorus in protein-free blood filtrates using ammonium molybdite first described by Fiske and Subbarow ^12^. Phosphate and phosphate esters are first reduced using ferrous ammonium sulfate, and the absorbance measured at 340 nm. | OSR6222 |
| Total Protein | This Total Protein procedure is based on the modification of Weichselbaum.2 Cupric ions in an alkaline solution react with proteins and polypeptides containing at least two peptide bonds to produce a violet colored complex. The absorbance of the complex at 540/660 nm is directly proportional to the concentration of protein in the sample ^17^ | OSR6632 |
| Potassium | Potassium is measured using an ion selective electrode. |  |
| Sodium | Sodium is measured using an Ion Selective electrode. |  |
| Triglycerides | This Triglyceride procedure is based on a series of coupled enzymatic reactions.^18,19^ The triglycerides in the sample are hydrolyzed by a combination of microbial lipases to give glycerol and fatty acids. The glycerol is phosphorylated by adenosine triphosphate (ATP) in the presence of glycerol kinase (GK) to produce glycerol-3-phosphate. The glycerol-3-phosphate is oxidized by molecular oxygen in the presence of GPO (glycerol phosphate oxidase) to produce hydrogen peroxide (H2O2) and dihydroxyacetone phosphate. The formed H2O2 reacts with 4-aminophenazone and N,N-bis(4-sulfobutyl)-3,5-dimethylaniline, disodium salt (MADB) in the presence of peroxidase (POD) to produce a chromophore, which is read at 660/800nm. The increase in absorbance at 660/800 nm is proportional to the triglyceride content of the sample. | OSR66118 |
| Urea | This Urea Nitrogen procedure is based on an adaptation of the enzymatic method of Talke and Schubert.^20^ In this method, urea is hydrolyzed enzymatically by urease to yield ammonia and carbon dioxide. The ammonia and α-oxoglutarate are converted to glutamate in a reaction catalyzed by L-glutamate dehydrogenase (GLDH). Simultaneously, a molar equivalent of reduced NADH is oxidized.^3,4,5^ Two molecules of NADH are oxidized for each molecule of urea hydrolyzed. The rate of change in absorbance at 340 nm, due to the disappearance of NADH, is directly proportional to the BUN concentration in the sample. | OSR6634 |
| Uric Acid | This Uric Acid procedure is a modification of the Fossati method. Uric acid is converted by uricase to allantoin and hydrogen peroxide. Hydrogen peroxide reacts with 4-aminoantipyrine (4-AAP) in the presence of N,N-bis(4-sulfobutyl)-3,5-dimethylaniline, disodium salt (MADB) to produce a chromophore which is read bichromatically at 660/800 nm. The amount of dye formed is proportional to the uric acid concentration in the sample.^21^ | OSR6698 |

1. Rodkey, F.L., Clin Chem, 2: 478; 1965.

2. Doumas, B.T., Watson, W.A. and Biggs, H.G., Clin Chem Acta 31: 87-96, 1971.

3. Bowers, G.N., and McComb, R.B., Clin Chem. 21: 1988 -1995, 1975

4. International Federation of Clinical Chemistry. Clin Chem; 23: 887, 1977.

5. Ehrlich, P., Charite-Annalen, 8: 140, 1883.

6. Bauer, P.J.: Anal. Biochem., 110: 61, 1981.

7. Michaylova, V.; Ilkova, P.: Anal. Chim. Acta, 53: 194, 1971.

8. Allain CC, Poon LS, Chan CSG, Richmond W, Fu PC. Enzymatic determination of total serum cholesterol. Clin Chem 1974;20:470-475.

9. Horder M., Elsner R., *et al*., Approved Recommendation of IFCC Methods for the Measurement of Catalytic Concentration of Enzymes, Part 7 IFCC Method for Creatine Kinase. J. Clin. Chem. Clin. Biochem. 29, 435, 1991.

10. Szasz, G., Gerhardt, W. and Gruber, W., Clin Chem, 23: 1888, 1977.

11. Jaffe, M.Z. Physiol Chem, 10: 391,1886.

12. Fiske, C.H., Subbarow, Y., J Biol Chem, 66: 375, 1925.

13. Wacker, W. E. C., Ulmer, D.D. and Vallee, B.L., N. Eng J Med, 255: 449, 1956.

14. Amador, E., Dorfman, L. E. and Wacker, W.E.C., Clin Chem, 9: 391, 1963.

15. Mann, C.K. and Yoe, J.H., Anal Chem, 28: 202-205, 1956.

16. Mann, C.K. and Yoe, J.H., Anal Chem Acta, 16: 155-160, 1957

17. Weichselbaum, T.E., Amer J Clin Path, 16: 40, 1946.

18. Trinder, P., Ann Clin Biochem, 6: 24, 1969.

19. Bucolo, G. and David, H., Clin Chem, 19: 476, 1973.

20. Talke, H. and Schubert, G.E., Klinische Wochenschrift, 43: 174 1965.

21. Fossati, P., Prencipe, L. and Berti, G., Clin Chem, 26: 227, 1980.

## References

**Aguirre AA and Balazs GH** (2000) Blood biochemistry values of green turtles, *Chelonia mydas*, with and without fibropapillomatosis. Comp. Haematol. Int., 10(3), 132-

137. doi:10.1007/s005800070004

**Aguirre AA, Balazs GH, Spraker TR, Gross TS** (1995) Adrenal and hematological responses to stress in juvenile green turtles (*Chelonia mydas*) with and without

fibropapillomas. Physiol. Zool., 68(5), 831-854. doi:10.1086/physzool.68.5.30163934

**Anderson ET, Harms CA, Stringer EM, Cluse WM** (2011) Evaluation of hematology and serum biochemistry of cold-stunned green sea turtles (*Chelonia mydas*) in North

Carolina, USA. J. Zoo Wildl. Med., 42(2), 247-255. doi:10.1638/2010-0217.1

**Arthur KE., Limpus CJ, Whittier JM** (2008) Baseline blood biochemistry of Australian green turtles (*Chelonia mydas*) and effects of exposure to the toxic

cyanobacterium *Lyngbya majuscula*. Aust. J. Zool., 56(1), 23-32. doi:10.1071/zo08055

**Bloodgood JCG, Norton TM, Hoopes LA, Stacy NI, Hernandez SM** (2019) Comparison of hematological, plasma biochemical, and nutritional analytes of rehabilitating

and apparently healthy free-ranging Atlantic green turtles (*Chelonia mydas*). J. Zoo Wildl. Med., 50(1), 69-81. doi:10.1638/2017-0250

**Bolten AB and Bjorndal KA** (1992) Blood profiles for a wild population of green turtles (*Chelonia mydas*) in the southern Bahamas: Size-specific and sex-specific

relationships. J. Wildl. Dis., 28(3), 407-413.

**de Mello DMD, Alvarez MCL** (2020) Health assessment of juvenile green turtles in southern Sao Paulo state, Brazil: A hematologic approach. J. Vet. Diagn. Invest., 32(1),

25-35. doi:10.1177/1040638719891972

**Flint M, Brand AF, Bell IP, Hof CAM** (2019) Monitoring the health of green turtles in Northern Queensland post catastrophic events. Sci. Total Environ., 660, 586-592.

doi:10.1016/j.scitotenv.2019.01.065

**Flint M, Morton JM, Limpus CJ, Patterson-Kane JC, Murray PJ Mills PC** (2010) Development and application of biochemical and haematological reference intervals

to identify unhealthy green sea turtles (*Chelonia mydas*). The Veterinary Journal, 185(3), 299-304. doi:10.1016/j.tvjl.2009.06.011

**Fong C-l, Chen H-C, Cheng I-J** (2010) Blood profiles from wild populations of green sea turtles in Taiwan. Journal of Veterinary Medicine and Animal Health, 2(2), 008-

010.

**Fuenmayor AJM, Rangel JLH, Arraga MCA** (2006) Hematological values of the green turtle (*Chelonia mydas*) present in the High Guajira. Rev. Cient.-Fac. Cienc. Vet.,

16(3), 219-226.

**Hamann M, Schäuble CS, Simon T, Evans S** (2006) Demographic and health parameters of green sea turtles *Chelonia mydas* foraging in the gulf of Carpentaria, Australia.

Endangered Species Research, 2, 81-88.

**Hasbun CR, Lawrence AJ, Naldo J, Samour JH, Al-Ghais SM** (1998) Normal blood chemistry of free-living green sea turtles, *Chelonia mydas*, from the United Arab

Emirates. Comp. Haematol. Int., 8(3), 174-177. doi:10.1007/bf02642510

**Hirama S, Ehrhart LM, Rea LD, Kiltie RA** (2014) Relating fibropapilloma tumor severity to blood parameters in green turtles *Chelonia mydas*. Dis. Aquat. Organ.,

111(1), 61-68. doi:10.3354/dao02765

**Kophamel S, Munns SL** (2022) Data from: Haematological and biochemical reference intervals for wild green turtles (*Chelonia mydas*): a Bayesian approach for small

sample sizes. James Cook University. [https://doi.org/10.25903/9rm7-k267](https://aus01.safelinks.protection.outlook.com/?url=https%3A%2F%2Fdoi.org%2F10.25903%2F9rm7-k267&data=05%7C01%7Csara.kophamel%40my.jcu.edu.au%7Ceede1e95d1a948edf97808da2270d11b%7C2eba4cf8af764db3bcaf81b5592535ef%7C0%7C0%7C637860165213286113%7CUnknown%7CTWFpbGZsb3d8eyJWIjoiMC4wLjAwMDAiLCJQIjoiV2luMzIiLCJBTiI6Ik1haWwiLCJXVCI6Mn0%3D%7C3000%7C%7C%7C&sdata=A%2Bo2iTbQbVM9xqnhZS5VaFTQh29%2FPu1K0rZyxXqJM%2FA%3D&reserved=0).

**Labrada-Martagon V, Mendez-Rodriguez LC, Gardner SC, Lopez-Castro M, Zenteno-Savin T** (2010) Health indices of the green turtle (*Chelonia mydas*) along the

Pacific coast of Baja California Sur, Mexico. I. Blood biochemistry values. Chelonian Conserv. Biol., 9(2), 162-172. doi:10.2744/ccb-0806.1

**Lara-Ucetal M, Hinojosa-Arango G, Aranda-Cirerol F, López-Vivas JM, Gutiérrez-Ruiz EG, Rousso S, Riosmena-Rodriguez R** (2016) Practical manual on clinical

cytology and hematology for sea turtle conservation. Advances in research techniques for the study of sea turtles, 165.

**Lewbart GA, Hirschfeld M, Denkinger J, Vasco K, Guevara N, García J, Muñoz J, Lohmann KJ** (2014) Blood gases, biochemistry, and hematology of Galapagos

green turtles (*Chelonia mydas*). PLoS One, 9(5), e96487. doi:10.1371/journal.pone.0096487

**Li TH and Chang CC** (2020) The impact of fibropapillomatosis on clinical characteristics, blood gas, plasma biochemistry, and hematological profiles in juvenile green

turtles (*Chelonia mydas*). Bull. Mar. Sci., 96(4), 723-734. doi:10.5343/bms.2019.0120

**March DT, Vinette-Herrin K, Peters A, Ariel E, Blyde D, Hayward D, Christidis L, Kelaher BP** (2018) Hematologic and biochemical characteristics of stranded green

sea turtles. J. Vet. Diagn. Invest., 30(3), 423-429. doi:10.1177/1040638718757819

**McNally KL, Mott CR, Guertin JR, Gorham JC, Innis CJ** (2020) Venous blood gas and biochemical analysis of wild captured green turtles (*Chelonia mydas*) and kemp's

ridley turtles (*Lepidochelys kempii*) from the Gulf of Mexico. PLoS One, 15(8), 11. doi:10.1371/journal.pone.0237596

**Montilla AJ, Hernandez JL, Bravo AR, Vera VJ, Mesa JF** (2008) Blood biochemistry values of green turtle (*Chelonia mydas*) present in the Alta Guajira, Venezuela.

Rev. Cient.-Fac. Cienc. Vet., 18(4), 351-357.

**Page-Karjian A, Chabot R, Stacy NI, Morgan AS, Valverde RA, Stewart S, Coppenrath CM, Manire CA, Herbst LH, Gregory CR** (2020) Comprehensive health

assessment of green turtles *Chelonia mydas* nesting in southeastern Florida, USA. Endangered Species Research, 42, 21-35.

**Page-Karjian A, Rivera S, Torres F, Diez C, Moore D, Van Dam R, Brown C** (2015) Baseline blood values for healthy free-ranging green sea turtles (*Chelonia mydas*) in

Puerto Rico. *Comparative Clinical Pathology* 24: 567-573

**Percie du Sert N, Ahluwalia A, Alam S, Avey MT, Baker M, Browne WJ, Clark A, Cuthill IC, Dirnagl U, Emerson M *et al.*** (2020) Reporting animal research:

Explanation and elaboration for the arrive guidelines 2.0. *PLoS Biology* 18: e3000411

**Perrault JR, Bresette MJ, Mott CR, Stacy NI** (2018) Comparison of whole blood and plasma glucose concentrations in green turtles (*Chelonia mydas*) determined using a

glucometer and a dry chemistry analyzer. J. Wildl. Dis., 54(1), 196-199. doi:10.7589/2017-08-198

**Prieto-Torres DA, Hernandez JL, Henriquez AR, Alvarado MC, Davila MJ** (2013) Blood biochemistry of the breeding population of green turtles (*Chelonia mydas*) in

the Aves Island Wildlife Refuge, Venezuela. South Am. J. Herpetol., 8(3), 147-154. doi:10.2994/sajh-d-13-00010.1

**Prieto-Torres DA, Hernandez-Rangel JL, Bravo-Henrique AR, Alvarado-Arraga MC, Davila-Ojeda MJ, Quiroz-Sanchez NR** (2012) Hematological values of the

nesting population of green turtles (*Chelonia mydas*) in the Wildlife Refuge Aves Island, Venezuela. Rev. Cient.-Fac. Cienc. Vet., 22(3), 273-280.

**Putillo AR, Flint M, Seminoff JA, Spencer RGM, Fuentes M** (2020) Plasma biochemistry profiles of juvenile green turtles (*Chelonia mydas*) from the Bahamas with a

potential influence of diet. J. Wildl. Dis., 56(4), 768-780. doi:10.7589/jwd-d-20-00009

**Reséndiz E, Fernández-Sanz H, Ramos D, Silva V, Lara-Ucetal M** (2018) Condition and haematological values of free-ranging eastern pacific green turtles (*Chelonia*

*mydas*) from Baja California Sur, Mexico. SM J Clin Pathol, 3(1), 1016.

**Rossi S, Zwarg T, Sanches TC, Cesar MD, Werneck MR, Matushima** ER (2009) Hematological profile of *Chelonia mydas* (testudines, cheloniidae) according to the

severity of fibropapillomatosis or its absence. Pesquisa Veterinaria Brasileira, 29(12), 974-978. doi:10.1590/s0100-736x2009001200004

**Samour JH, Howlett JC, Silvanose C, Hasbun CR, Al-Ghais SM** (1998) Normal haematology of free-living green sea turtles (*Chelonia mydas*) from the United Arab

Emirates. Comp. Haematol. Int., 8(2), 102-107. doi:10.1007/bf02642499

**Sinaei M, Bolouki M, Ghorbanzadeh SG, Matin MT** (2019) Evaluation of hematological and plasma biochemical parameters in green sea turtle (*Chelonia mydas*

*linnaeus*, 1758) from nesting colonies of the northern coast the Sea of Oman. Iran. J. Fish. Sci., 18(4), 891-902. doi:10.22092/ijfs.2019.118320

**Snoddy JE, Landon M, Blanvillain G, Southwood A** (2009) Blood biochemistry of sea turtles captured in gillnets in the lower cape fear river, North Carolina, USA. J.

Wildl. Manage., 73(8), 1394-1401. doi:10.2193/2008-472

**Stewart K, Norton T, Mohammed H, Browne D, Clements K, Thomas K, Yaw T, Horrocks J** (2016) Effects of “swim with the turtles” tourist attractions on green sea

turtle (*Chelonia mydas*) health in Barbados, West Indies. J. Wildl. Dis., 52(2s), S104-S117.

**Suarez-Yana T, Montes D, Zuniga R, Mangel JC, Alfaro-Shigueto J** (2016) Hematologic, morphometric, and biochemical analytes of clinically healthy green sea turtles

(*Chelonia mydas*) in Peru. Chelonian Conserv. Biol., 15(1), 153-157. doi:10.2744/ccb-1160.1

**Whiting SD, Guinea ML, Limpus CJ, Fomiatti K** (2007) Blood chemistry reference values for two ecologically distinct populations of foraging green turtles, Eastern

Indian Ocean. Comp. Clin. Path., 16(2), 109-118.

**Wood FE and Ebanks GK** (1984) Blood cytology and hematology of the green sea turtle, *Chelonia mydas*. Herpetologica, 40(3), 331-336.

**Work TM, Raskin RE, Balazs GH, Whittaker SD** (1998) Morphologic and cytochemical characteristics of blood cells from Hawaiian green turtles. Am. J. Vet. Res.,

59(10), 1252-1257.

**Zwarg T, Rossi S, Sanches TC, Cesar MD, Werneck MR, Matushima ER** (2014) Hematological and histopathological evaluation of wildlife green turtles (*Chelonia*

*mydas*) with and without fibropapilloma from the north coast of Sao Paulo state, Brazil. Pesquisa Veterinaria Brasileira, 34(7), 682-688. doi:10.1590/s0100-

736x2014000700013

1. ^¶^ These authors contributed equally to this work. [↑](#footnote-ref-1)
